# ATF3 drives senescence by reconstructing accessible chromatin profiles

**DOI:** 10.1111/acel.13315

**Published:** 2021-02-04

**Authors:** Chao Zhang, Xuebin Zhang, Li Huang, Yiting Guan, Xiaoke Huang, Xiao‐Li Tian, Lijun Zhang, Wei Tao

**Affiliations:** ^1^ The MOE Key Laboratory of Cell Proliferation and Differentiation School of Life Sciences Peking University Beijing China; ^2^ PKU‐Tsinghua‐NIBS Graduate Program School of Life Sciences Peking University Beijing China; ^3^ Department of Human Population Genetics Human Aging Research Institute (HARI) and School of Life Sciences Nanchang University Nanchang China

**Keywords:** AP‐1, ATF3, chromatin accessibility, IARs, DNA methylation, heterochromatin, DARs, senescence

## Abstract

Chromatin organization and transcriptional profiles undergo tremendous reordering during senescence. However, uncovering the regulatory mechanisms between chromatin reconstruction and gene expression in senescence has been elusive. Here, we depicted the landscapes of both chromatin accessibility and gene expression to reveal gene regulatory networks in human umbilical vein endothelial cell (HUVEC) senescence and found that chromatin accessibilities are redistributed during senescence. Particularly, the intergenic chromatin was massively shifted with the increased accessibility regions (IARs) or decreased accessibility regions (DARs), which were mainly enhancer elements. We defined AP‐1 transcription factor family as being responsible for driving chromatin accessibility reconstruction in IARs, where low DNA methylation improved binding affinity of AP‐1 and further increased the chromatin accessibility. Among AP‐1 transcription factors, we confirmed ATF3 was critical to reconstruct chromatin accessibility to promote cellular senescence. Our results described a dynamic landscape of chromatin accessibility whose remodeling contributes to the senescence program, we identified that AP‐1 was capable of reorganizing the chromatin accessibility profile to regulate senescence.

## Graphical abstract

Senescence‐specific increased accessibility regions (IARs) are involved in senescence regulation. The gene regulatory network is rewired during senescence, a process possibly determined by chromatin accessibility remodeling. DNA methylation is negatively correlated with chromatin accessibility, and loss of DNA methylation may help establish chromatin accessibility. The AP‐1 family, especially ATF3, is a potential regulator that remodels the accessibility in IARs in HUVEC senescence and that may be an effective target for senescence intervention.

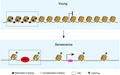

## 1 INTRODUCTION

Cellular senescence, an irreversible cell cycle arrest that triggers a series of progressive cellular states and phenotypic changes (van Deursen, 2014), is intricately involved in many biological processes including tumor suppression, embryogenesis, tissue repair, host immunity, aging, and age‐related disorders (He & Sharpless, 2017). Historically, senescence mechanisms have been viewed as replicative and stress‐induced (Hayflick & Moorhead, 1961; de Magalhaes & Passos, 2018). So, while the effects of senescence are well known, much is yet unknown about the upstream part of the regulatory network. Of the candidate hallmarks that may contribute to aging (Lopez‐Otin et al., 2013), epigenetic alterations, including spontaneous or passive changes, are most compelling.

The epigenome is a bridge that connects the genotype with the phenotype and that enables the interpretation of genomic information. Epigenetics is an extremely important contributor to senescence and aging (Sen et al., 2016) and includes alterations in DNA methylation patterns, posttranslational histone modification, and chromatin remodeling. During replicative senescence, demethylation of the entire genome has finished while local hypermethylation continues (Cruickshanks et al., 2013), and those DNA methylation patterns can be used to estimate the state of cellular senescence, a method called the DNA methylation clock (Horvath, 2013). Histone modifications reflect the different statuses of chromatin structures, and the varying patterns of those modifications enable the investigation of the senescence and aging regulatory mechanisms. Epigenetic modification examples include H3K4me3‐ or H3K27me3‐enriched mesas and H3K27me3‐depleted canyons in senescent cells, which are correlated with gene expression dynamics during senescence (Shah et al., 2013), spatial repositioning of H3K9me3 and H3K27me3 during senescence (Chandra et al., 2012), enrichment of H4K16ac in expressed genes in senescent cells (Rai et al., 2014), rearrangement of H3K27ac, which possibly promotes senescence (Sen et al., 2019; Tasdemir et al., 2016), and the loss of H4K20me3, which promotes senescence and aging (Lyu et al., 2018).

DNA methylation alterations and histone modifications change chromatin architecture in ways that affect gene expression profiles and thus determine cell fate. Previous researches showed that structural changes in chromatin are closely connected to senescence (Guan et al., 2020) and a chromatin remodeling procedure during senescence in nuclei involves the formation of senescence‐associated heterochromatin foci, where specific proliferation‐associated genes are repressed (Zhang et al., 2005). While local facultative heterochromatin is being constructed, there is a global decrease in constitutive heterochromatin in senescence—the hypothesized “heterochromatin loss model of aging” (Villeponteau, 1997). Many senescence‐associated chromatin remodeling factors have been discovered. Both silencing and overexpression of brahma‐related gene 1 (*BRG1*), which codes for the ATPase subunit in the SWI/SNF chromatin remodeling complex, can induce cellular senescence in both human and rat mesenchymal stem cells (MSCs) (Alessio et al., 2010). Loss of NuRD complex leads to aging‐related chromatin defects (Pegoraro et al., 2009). Recently, an RNAi screening of epigenetic proteins indicated that p300 drives senescence that is mediated by de novo super‐enhancer formation (Sen et al., 2019). Because the results of this research have shown a close correlation between the rearrangement of chromatin architecture and senescence, we can illustrate the mechanism of senescence and aging from the view of chromatin landscape.

Through active DNA regulatory elements, such as enhancers and promoters, chromatin accessibility is important in regulating gene expression. The redistribution of accessible chromatin reflects the dynamic physical interactions between chromatin‐binding factors and DNA, which cooperate to regulate the gene expression profiles (Klemm et al., 2019). Analyses of chromatin accessibility have revealed dynamic gene regulatory networks in various physiological and pathological processes (Corces et al., 2018). Those research results indicate that chromatin accessibility may potentially predetermine the stimuli response program and even cell fate. AP‐1 transcription factors, including members of the ATF, Fos, and Jun families, participate in a wide range of cellular processes, such as proliferation and apoptosis (Shaulian & Karin, 2001). Mounting evidence indicates that AP‐1 is responsible for the establishment and maintenance of open chromatin orchestration with gene expression in multiple cell types, and thus functions partially as a pioneer factor (Vierbuchen et al., 2017).

Here, we describe the chromatin accessibility landscape during senescence in human umbilical vein endothelial cells (HUVECs) by using assay for transposase‐accessible chromatin sequencing (ATAC‐seq), a powerful tool that reveals genome‐wide chromatin accessibility (Buenrostro et al., 2013). Meanwhile, we integrated the open chromatin landscape and the gene expression profile to study the gene regulatory network during senescence and found that gene expression and chromatin accessibility undergo reprogramming in senescence. Accessibility of certain senescence‐related chromatin regions may be either gradually increased or decreased during senescence, which we called IARs or DARs, respectively. During senescence, IAR‐related signaling pathways contribute to senescence progression, but DAR‐related signaling pathways are mostly involved in physiological functions. Furthermore, IARs are enriched for enhancer elements, and DNA methylation levels of IARs are negatively correlated with chromatin accessibilities. We also found that AP‐1 transcription factors regulate the openness of IARs, and an AP‐1 member, ATF3, promotes cellular senescence by reprogramming chromatin accessibility.

## 2 RESULTS

### 2.1 Genome‐wide mapping of the transcriptional landscape in HUVEC replicative senescence

**FIGURE 1:**
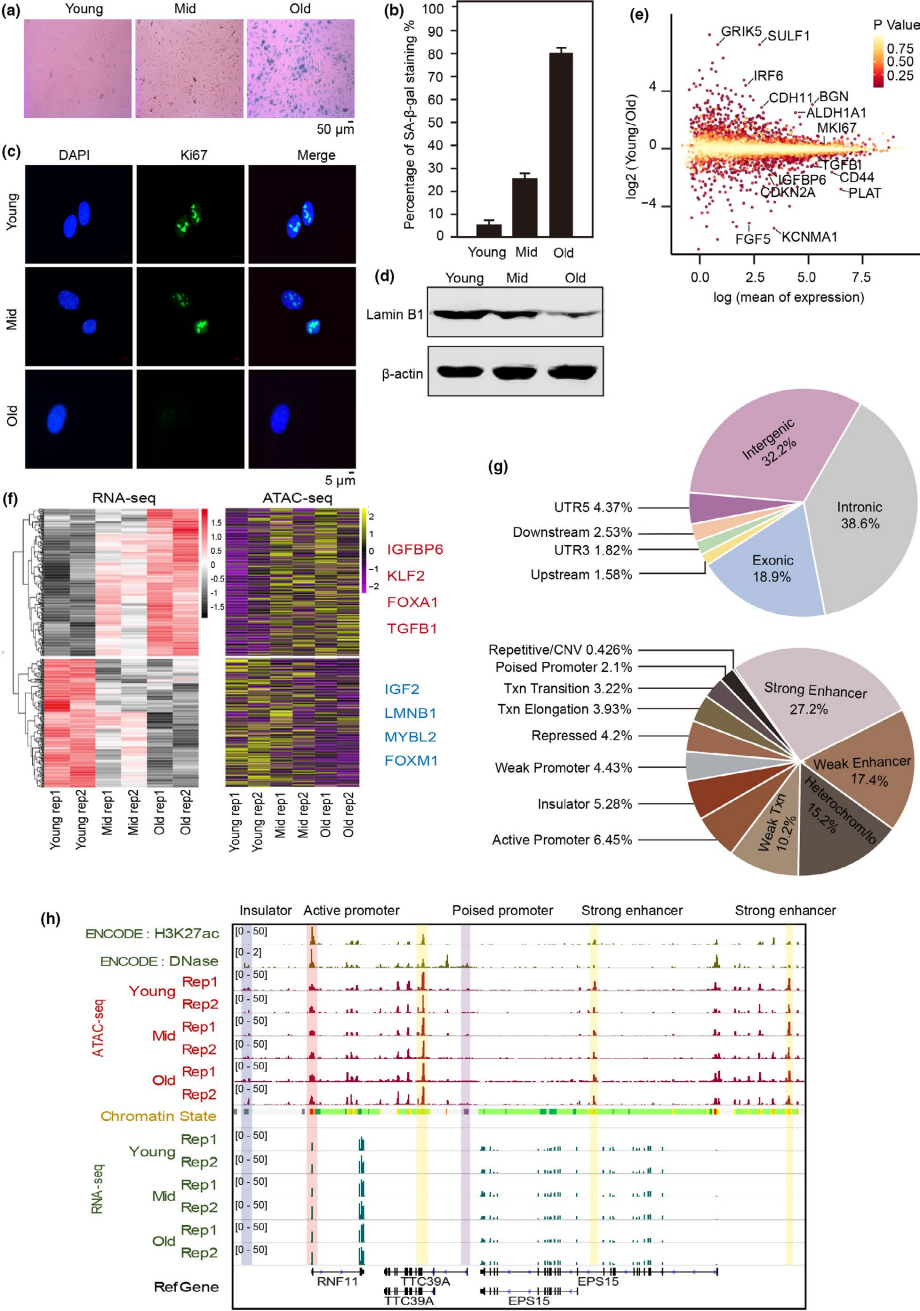
The genome‐wide landscape of gene expression and chromatin accessibility for replicative senescence. (a) SA‐β‐gal (senescence marker) stained young (PD8), middle‐aged (PD24), and old (PD36) human umbilical endothelial vein cells (HUVECs). PD, population doublings. (b) SA‐β‐gal‐positive HUVECs increased with number of passages. (c) Immunofluorescence of representative Ki67‐stained, serially passaged HUVECs. (d) Western blots showing Lamin B1 expression during HUVEC senescence. β‐Actin served as the loading control. (e) Dispersion plots of RNA‐seq signals showing the changes in selected genes’ expression between young and old HUVECs. (f) Heatmaps of HUVEC RNA‐seq and ATAC‐seq data sets at 3 age points with genes grouped according to similar gene expression patterns. Genes that were consistent between expression and promoter accessibility during senescence are listed to the right. Two independently repeated experiments were performed in each category (rep 1 and 2). (g) Annotations of all open chromatin regions. The upper pie chart shows the genomic features for the open chromatin regions, and the lower one shows the chromatin states. These results were generated from all ATAC‐seq peaks of three samples (young, mid, old). CNV, copy‐number variation; Txn, transcribed region; UTR, untranslated region. (h) Snapshot showing the signals of RNA‐seq, ATAC‐seq, H3K27ac, and DNase‐seq in example regions. The vertical gray box highlights the ATAC‐seq signals in insulator, enhancer and promoter. Chromatin states were obtained from ENCODE (light yellow = weak/poised enhancer, dark yellow = strong enhancer, green = transcribed region, blue = insulator, and gray = heterochromatin). Two biological replicates were performed of each condition (rep 1 and 2)

To decode the mechanism of senescence, we used primary HUVECs to build a replicative senescence system *in vitro*, in which HUVEC proliferation gradually slowed with each passaging and cultured growth was maintained for about 40 population doublings (about 20–30 passages) (Figure S1). Accordingly, we defined the population doubling 8 cells as young, the population doubling 24 cells as mid (middle‐aged), and the population doubling 36 cells as old. We also used senescence markers to better evaluate the reliability of our replicative system. SA‐β‐gal and Ki‐67 are classical senescence or proliferation markers, respectively (Dimri et al., 1995; Gerdes et al., 1987), and their signals gradually increased and then decreased, respectively, as HUVEC culture time progressed (Figure 1a‐c). In addition, Lamin B1, a molecular senescence marker whose protein level declines in most senescence systems (Freund et al., 2012), was down‐regulated in our system (Figure 1d). Together, we established a stable and reliable HUVEC senescence system.

Next, we used RNA‐seq to investigate dynamic changes in gene expressions in our HUVEC senescence model (Figure S2). First, the expressions of *CDKN1A*, *CDKN2A*, and *TGFBI* were gradually increased, while *LMNB1* and *MKI67* were gradually decreased during senescence (Figure 1e, Figure S2), consistent with previous studies (Freund et al., 2012; Lyu et al., 2018; Marthandan et al., 2016). Furthermore, gene ontology (GO) analysis showed that up‐regulated genes are mainly involved in the focal adhesion signaling pathway and extracellular matrix–receptor pathway, which are closely related to senescence phenotypes, such as adhesion plaque formation and cell migration (Figure S3a) (Borghesan & O'Loghlen, 2017). GO also showed that genes involved in the DNA replication‐ and cell cycle‐associated pathways were down‐regulated, results that conform with the definition of cell cycle arrest during senescence (Marthandan et al., 2016; Figure S3b).

### 2.2 The landscape of chromatin accessibility during senescence

ATAC‐seq enabled us to explore how the gene expression profile is programmed and to study the changes in chromatin accessibility during senescence. The distribution of fragment insertion size showed clear periodicity of ~200 bp, consistent with former study (Figure S4a) (Buenrostro et al., 2013). We proved the reliability of our ATAC‐seq data by comparing it with the public Encyclopedia of DNA Elements (ENCODE) DNase‐seq data (Figure S4b). ATAC‐seq signals were highly enriched at transcription start sites (TSS) (Sun et al., 2019), and alterations in the gene expression profile were accompanied by promoter accessibility redistribution (Figure S4c,d). And the gene expression levels were correlated with its promoter accessibilities during senescence. For example, *IGFBP6* (Coppe et al., 2010), *KLF2* (Taniguchi et al., 2012), and *TGFB1* (Senturk et al., 2010) expressions were highly up‐regulated during senescence and the genes’ promoter accessibilities were remarkably increased, while significantly down‐regulated genes, *IGF2* (DeChiara et al., 1990), *FOXM1* (Smirnov et al., 2016), and *MYBL2* (Musa et al., 2017) exhibited decreased chromatin accessibilities (Figure 1f). These results indicated that chromatin accessibility contributed to gene expression changes during senescence, especially in the key genes that may trigger senescence.

We next comprehensively characterized chromatin accessibility regions during senescence. We found that they were located mostly in the intergenic, intronic, and exonic regions, where enhancers were proportionally most abundant (Figure 1g). For example, ATAC‐seq signals were enriched in active promoters and strong enhancers around *RNF11* (Figure 1h). Altogether, our chromatin accessibility landscape revealed that ATAC‐seq peaks from HUVEC cells were the most prevalent located in enhancers.

### 2.3 Dynamics of chromatin accessibility during senescence

**FIGURE 2:**
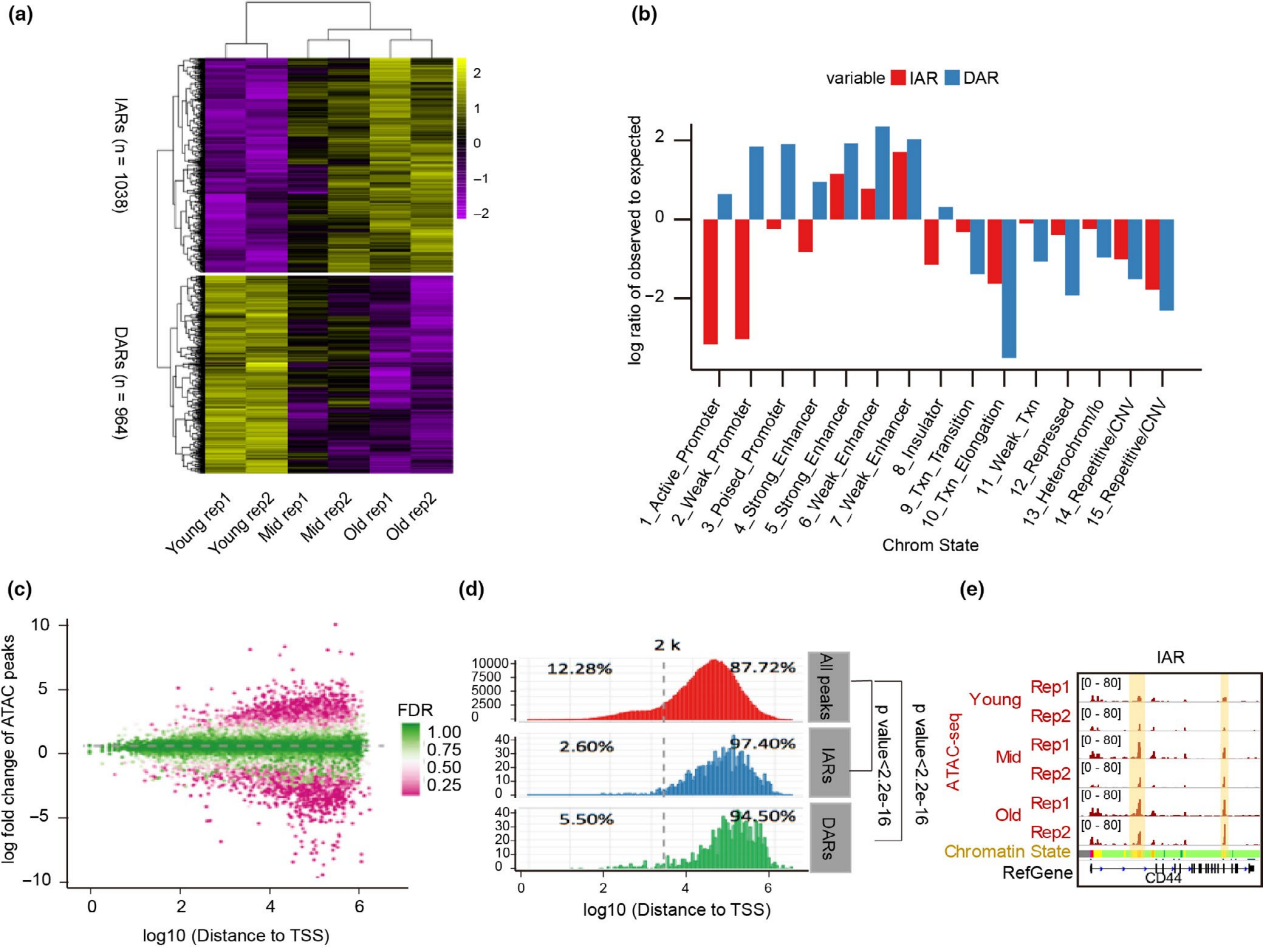
Dynamics of chromatin accessibility during senescence. (a) Heatmap of hierarchically clustered ATAC‐seq signals showing the accessibility remodeling of senescence‐related chromatin regions with increased accessibility (IARs) and senescence‐related chromatin regions with decreased accessibility (DARs) during senescence. Two biological replicates were performed in each category (rep 1 and 2). (b) Enrichment of chromatin states at IARs and DARs. (c) Scatter diagram showing alterations of ATAC‐seq signals between young and old cells in all peaks and with different distances to transcription start sites (TSSs). Most of the regions that changed markedly in senescence are far from their TSSs. FDR, false discovery rate. (d) The distributions of the distances between ATAC‐seq peaks (all peaks, IARs and DARs) and the TSSs. (e) Signal distributions of ATAC‐seq signals, DNase‐seq, and H3K27ac in or near the TMCO5A during HUVEC senescence. The vertical yellow box shows the gradually increased ATAC‐seq signals in annotated enhancer regions. See Figure 1 for senescence stage definitions and chromatin state information

After revealing the chromatin accessibility landscape during senescence, we compared the whole‐genome ATAC‐seq signals at different senescence stages to investigate senescence‐specific regions with chromatin accessibility changes, subsequently identifying almost 100,000 ATAC‐seq peaks per sample. Those peaks were dynamic in different senescence stages (Figure S5a), indicating that chromatin accessibility rearranged during senescence. By comparing ATAC‐seq peaks with public chromatin states, we found that the peaks were mainly located in enhancers (Figure S5b,c), indicating that the enhancer‐gene regulatory networks were reconstructed as senescence progressed. Next, we examined those chromatin regions that sustained accessibility remodeling during senescence and found 1038 increased accessibility regions (IARs) and 964 decreased accessibility regions (DARs) during senescence (Figure 2a). Remarkably, IARs are evolutionarily conserved (Figure S6), implying that they are regulatory elements.

To comprehensively understand the function of IARs or DARs, we compared them with public genomic elements and found that enhancer elements were enriched in IARs and DARs (Figure 2b) and that the changing accessibility regions were far from TSSs (Figure 2c). Most IARs (97.4%) and DARs (94.5%) were located 2 kb away from the TSS (Figure 2d), indicating they are potential enhancer elements. For example, the IARs in *CD44* were located in annotated enhancer elements (Figure 2e). Additionally, we found about 30%–40% peaks of IARs/DARs were distributed in the heterochromatin regions (Figure S7a), as illustrated by an IAR located near the *TMCO5* gene locus that was formerly annotated as heterochromatin and that also lacked H3K27ac and DNase‐seq signals (Figure S7b–e). This demonstrates that parts of heterochromatin regions were opening during HUVEC senescence.

Altogether, these results revealed that the chromatin accessibility profile was rearranged during senescence and that IARs were mainly distributed in enhancers.

### 2.4 Gene regulatory network rewires during senescence

**FIGURE 3:**
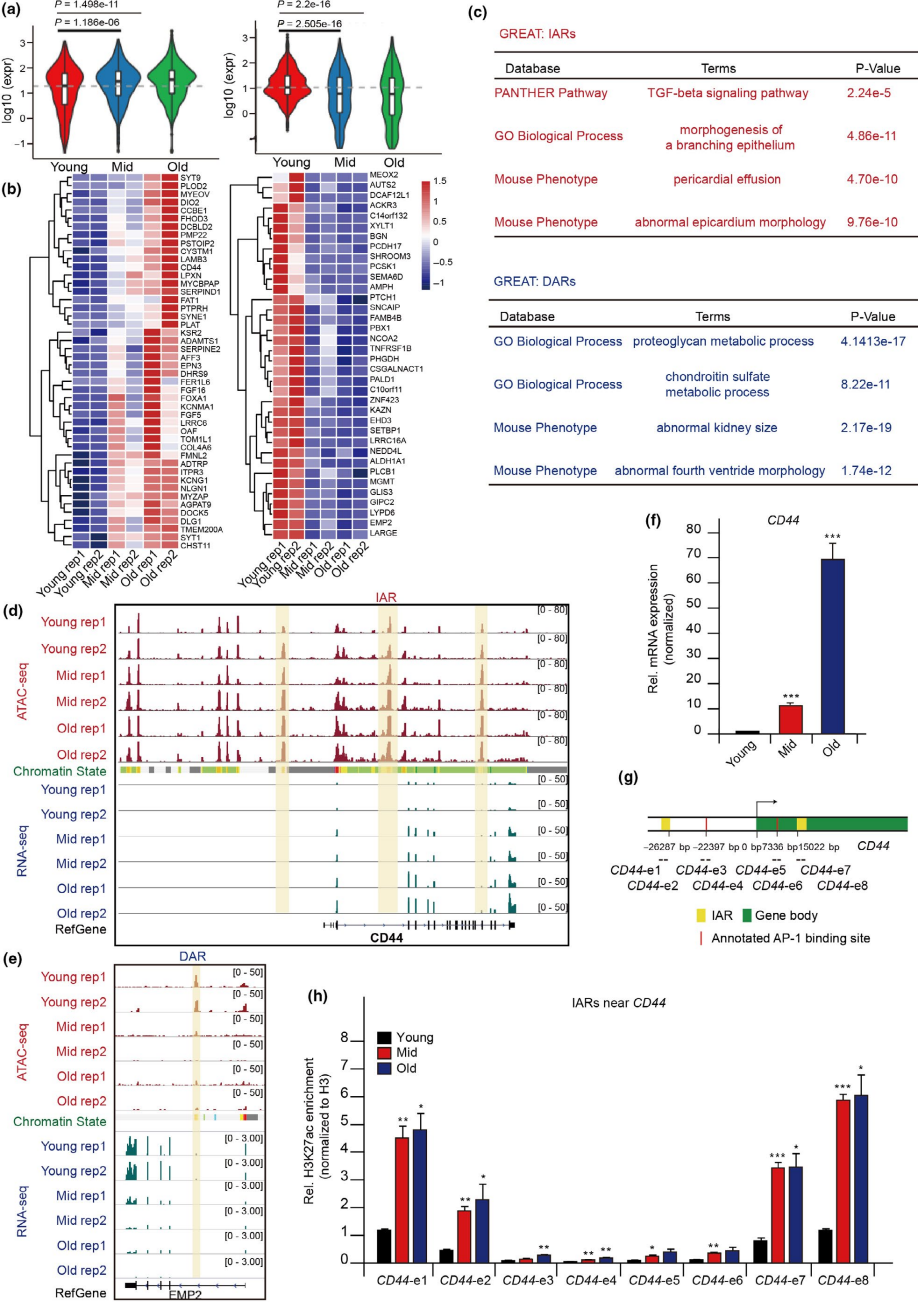
Gene regulatory network rewires during senescence. (a) Distributions of expression changes during senescence of genes located near IARs (left) or DARs (right). The *y*‐axis represents log‐transformed RNA‐seq signals, and the *x*‐axis represents the three HUVEC culture stages (defined in Figure 1a). (b) Heatmap of the hierarchically clustered expression of well‐differentiated genes belonging to IARs (left) and DARs (right). Two biological replicates were performed in each category (rep 1 and 2) (c) Genomic Regions Enrichment of Annotations Tool (GREAT) analysis of the functional enrichment of IARs and DARs. (d, e) ATAC‐seq and RNA‐seq signals at IARs near *CD44* (d) and DARs near *EMP2* (e). The vertical yellow boxes show the IARs and DAR. (f) RT‐qPCR results of *CD44* expression during HUVEC senescence. The cycle threshold (Ct) of *CD44* was normalized with *ACTB*. (g) Location information of the ChIP‐qPCR primers relative to *CD44*. TSS, transcription start site. (h) ChIP‐qPCR results of H3K27ac levels at the IARs in (g). The *y*‐axis represents the normalized H3K27ac signals relative to input, which normalized to H3. The error bars represent the s.d. obtained from triplicate independent experiments. Two‐tailed, unpaired Student's *t* tests were performed. *p < 0.05, **p < 0.01, and ***p < 0.001. See Figure 1 for senescence stage definitions and chromatin state information

To investigate how IARs and DARs function in the senescence fate decision, we analyzed the expression of genes found near IARs and DARs. We found that genes near IARs are up‐regulated, and genes near DARs are down‐regulated (Figure 3a,b). This result indicated that IARs and DARs may positively regulate the expression of neighboring genes, consistent with previous studies (Basisty et al., 2020; Hernandez‐Segura et al., 2017; Mun & Boo, 2010). The signaling pathways of the genes near IARs are involved in the TGF‐β pathway (Figure 3c), which is reported to be activated during senescence (Lyu et al., 2018). However, the dominant functions of genes near DARs are in proteoglycan and chondroitin metabolic processes. Further analyses of the disorders of related genes showed that perturbed IARs may cause pericardial effusion and epicardial morphological abnormalities (Figure 3c). Our results indicated that IARs may regulate senescence fate through senescence‐related signaling pathways that related genes are involved in.

For example, *CD44*, a senescence‐induced cell adhesion gene (Mun & Boo, 2010), is up‐regulated during HUVEC senescence and three IARs annotated as enhancer elements are near *CD44*, indicating that IARs may function as enhancers to regulate nearby genes’ expressions (Figure 3d). While we identified an IAR near *DIO2* (Figure S8a), Medeiros Tavares Marques et al. (2017) had found that *DIO2* is highly expressed in the hMSC senescence system, suggesting that *DIO2* may also be a senescence marker. Meantime, Jang et al. reported that *EMP2*, which regulates vascular endothelial growth factor A, is down‐regulated during senescence (Jang et al., 2015) and we found a DAR in *EMP2*, whose expression gradually descended during senescence (Figure 3e). Our study identified seven DARs near *SULF1*, and its expression also gradually decreased with senescence (Figure S8b). Our results indicated that the IARs were associated with enhancers and the reconstruction of chromatin accessibility at IARs may regulate target genes’ expression during senescence.

Furthermore, we used both real‐time (RT)‐ and chromatin immunoprecipitation (ChIP)‐qPCR methods to verify the expression levels and the IARs’ enhancer activities of adjacent genes. We found elevated mRNA levels of *CD44* and other genes (Figure 3f and Figure S9c,d). Correspondingly, ChIP‐qPCR signals of H3K27ac and H3K4me1, the epigenetic markers of enhancer activities, gradually increased during senescence at IARs near *CD44* and other genes and ATAC‐seq peaks (Figure 3g,h, and Figure S9). Those findings showed that IARs serve as epigenetic enhancers that may regulate nearby genes’ expressions. In summary, reconstruction of chromatin accessibility affects enhancer activities in senescence‐specific IARs and DARs, which may further regulate the expression of adjacent senescent genes.

### 2.5 DNA methylation may contribute to establish chromatin accessibility

**FIGURE 4:**
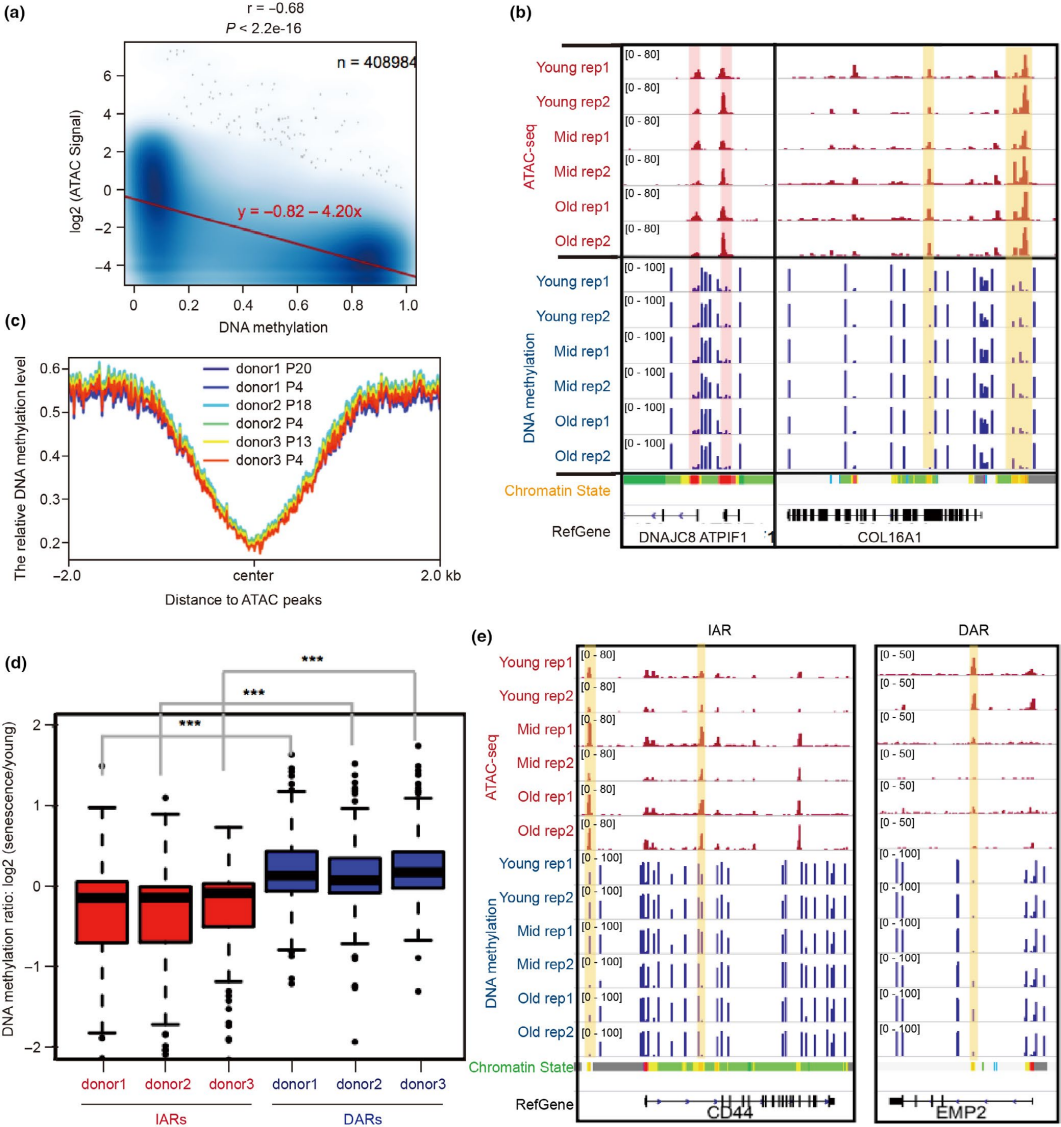
DNA methylation may help establish chromatin accessibility. (a) The correlation of chromatin accessibility and DNA methylation in all ATAC‐seq peaks during HUVEC senescence. (b) ATAC‐seq and DNA methylation signals near *DNAJC8*, *ATPIF1*, and *COL16A1*. The red and yellow vertical boxes show the opposite trend between ATAC‐seq and DNA methylation signals. (c) Distribution of DNA methylation signals around ATAC peaks. (d) The box plots showing DNA methylation level changes at IARs and DARs during senescence. The HUVECs were donated from three volunteers: donor 1, donor 2, and donor 3. The y‐axis represents the logarithm ratio of 5‐methylcytosine (5mC) levels in senescent cells to that in young cells. Two‐tailed, unpaired Student's *t* tests were performed. ***p < 0.001. (e) Changes in DNA methylation and ATAC‐seq signals in the IARs near *CD44* (left) and the DAR near *EMP2* (right) during senescence. The vertical yellow boxes show IARs and DARs. For (b) and (e), 2 independently repeated experiments were performed in each category (rep 1 and 2). See Figure 1 for senescence stage definitions and chromatin state information

The process of overall chromatin accessibility undergoing regular rearrangement during senescence is very similar to the reconstruction pattern of DNA methylation during senescence (Choy et al., 2010). So, we wondered whether there is a relationship between DNA methylation and chromatin accessibility during senescence. To answer this question, we first compared data of DNA methylation (5‐methylcytosine) in HUVEC senescence (Franzen et al., 2017) with our ATAC‐seq data, and found that ATAC‐seq signals were negatively correlated with DNA methylation levels during senescence (Figure 4a) and that DNA methylation was low at ATAC‐seq peak regions (Figure 4b,c). Those findings were consistent with previous studies that showed DNA methylation is depleted at promoter and enhancer regions (Schmidl et al., 2009). Therefore, chromatin accessibility and DNA methylation oppose each other at regulatory elements, such as the promoter regions of *DNAJC8* and *ATPIF1* and the enhancers of *COL16A1*, where ATAC‐seq signals are enriched, but DNA methylation signals are depleted (Figure 4b).

We continued to explore the dynamic changes between DNA methylation and chromatin accessibility during senescence by analyzing the changes of DNA methylation in IARs and DARs. We found that DNA methylation levels gradually declined in IARs and gradually rose in DARs during senescence (Figure 4d). For instance, along with senescence, ATAC‐seq signals gradually increased, while DNA methylation signals gradually decreased in IARs of *CD44*. However, the changes were opposite in the DAR of *EMP2* as ATAC‐seq signals gradually decreased, while DNA methylation signals gradually increased along with senescence (Figure 4e). In summary, our results indicated that the re‐establishment of DNA methylation patterns was involved in chromatin accessibility remodeling. Decreasing DNA methylation may contribute to chromatin accessibility at regulatory elements.

### 2.6 AP‐1 promotes senescence by regulating chromatin accessibility in IARs

**FIGURE 5:**
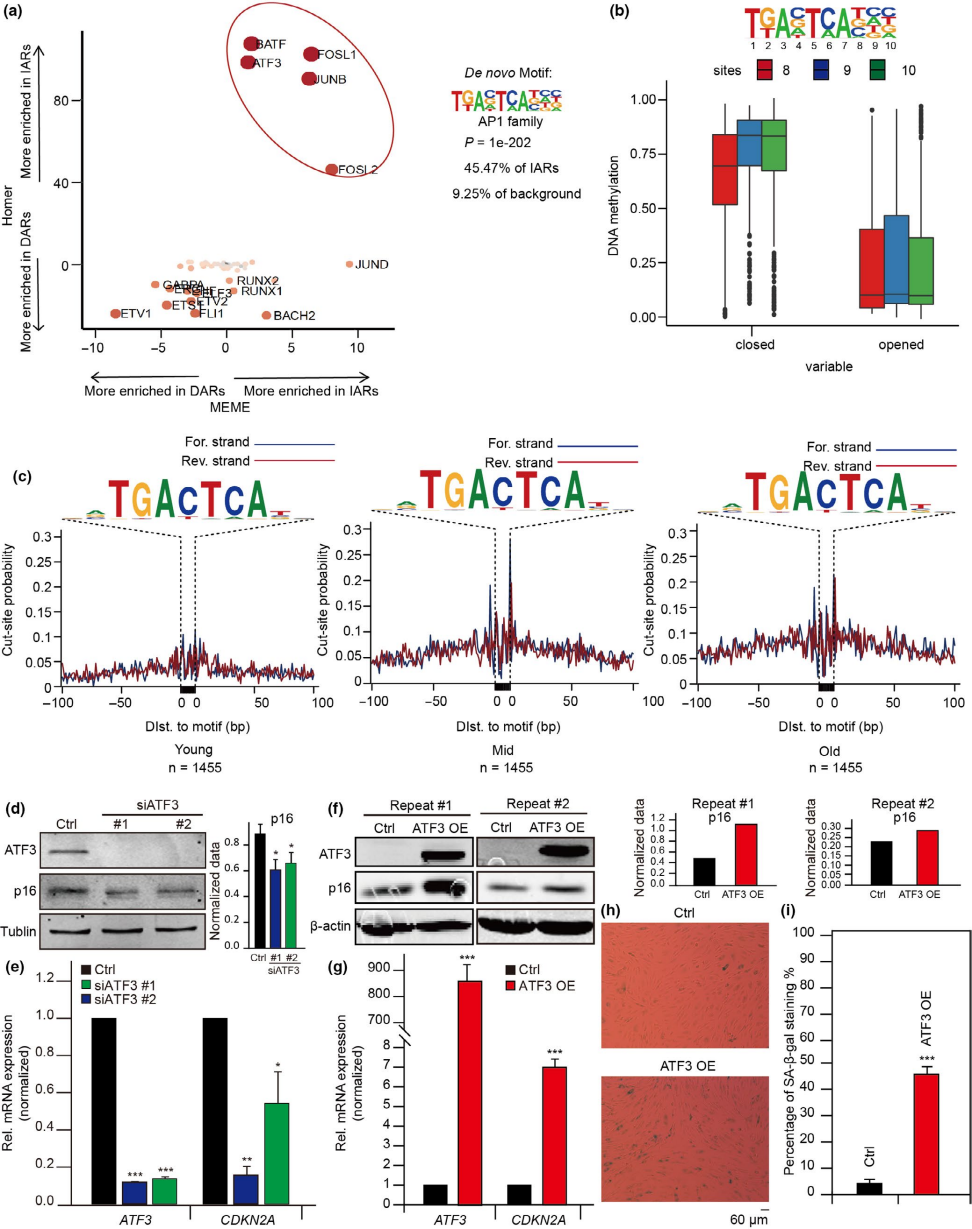
AP‐1 promotes senescence by regulating the accessibility of IARs. (a) Comparison of HOMER and MEME motif enrichment analyses of transcription factors at IARs and DARs. (b) Comparisons of DNA methylation at AP‐1 motif sites 8, 9, and 10 for both closed and open chromatin. (c) ATAC‐seq footprint analyses of ATF3 as HUVEC senescence. See Figure 1 for senescence stage definitions. (d) Western blots showing ATF3 and p16 protein levels after knocking down *ATF3* with its siRNA. Tubulin served as the loading control. The gray value of p16 blots was quantified by ImageJ 1.52a and normalized to tubulin (*n* = 3 experiments, *p < 0.05). (e) RT‐qPCR assay for the mRNA levels of *ATF3* and *CDKN2A* after knocking down *ATF3* with its siRNA. The cycle threshold (Ct) of *ATF3* and *CDKN2A* was normalized with *ACTB*. (f) The proteins levels of ATF3 and p16 in *ATF3*‐overexpressed HUVECs measured by Western blots. β‐Actin served as the loading control. OE, overexpression. The gray value of p16 blots was quantified by ImageJ 1.52a and normalized to β‐actin. (g) The mRNA expression of *ATF3* and *CDKN2A* in ATF3‐overexpressed HUVECs measured by RT‐qPCR. The cycle threshold (Ct) of *ATF3* and *CDKN2A* was normalized with *ACTB*. (h) SA‐β‐gal staining assay shows the difference of senescence phenotypes in ATF3‐overexpressed and in control HUVECs. (i) Statistical comparison of SA‐β‐gal‐positive cells in (h). The error bars represent the s.d. obtained from triplicate independent experiments. Two‐tailed, unpaired Student's *t* tests were performed. *p < 0.05, **p < 0.01, and ***p < 0.001

To get at key factors that may drive chromatin accessibility remodeling, we used two algorithms (HOMER and MEME) for motif enrichment analysis of transcription factors in IARs and DARs and found that the AP‐1 family was the most enriched in IARs (Figure 5a, Figure S10a,b). Among the members of AP‐1 family, ATF3’s motif was the most significant one (Figure S10d). Compared with the remarkable enrichment of transcription factors in IARs, those motifs enriched in DARs were less notable, whereas *ETV1*, *ETS*, *GABPA*, *ERG*, and *FLI1* were the most prevalent (Figure 5a, Figure S10a,b). Also, ATAC‐seq signals at AP‐1 motifs decreased in DARs during senescence, an opposite effect to that in IARs (Figure S10c). Combined with the classic senescence signaling pathways that may be regulated by IARs (Figure 3c), the AP‐1 family likely drives HUVEC senescence by remodeling chromatin accessibility at IARs. Moreover, the methylation levels at AP‐1 motifs were lower in open than in closed chromatin regions (Figure 5b), indicating that DNA methylation levels in AP‐1 may affect IARs’ chromatin accessibility.

Among the AP‐1 transcription factors enriched in IARs, the highest ranked, ATF3, attracted our attention. We found that ATF3 footprints gradually appeared as senescence progressed (Figure 5c), indicating that ATF3 could regulate the accessibility in IARs. So, we performed *ATF3* knockdown and overexpression experiments in HUVECs and found that after ATF3 defection, the expression of p16, a marker of senescence, was down‐regulated (Figure 5d,e). And the SA‐β‐gal signal was reduced after knocking down *ATF3* (Figure S15c,d). Conversely, when ATF3 was overexpressed, the p16 expression was up‐regulated and the SA‐β‐gal signal was enhanced (Figure 5f–i), thus demonstrating that *ATF3* can indeed promote senescence.

**FIGURE 6:**
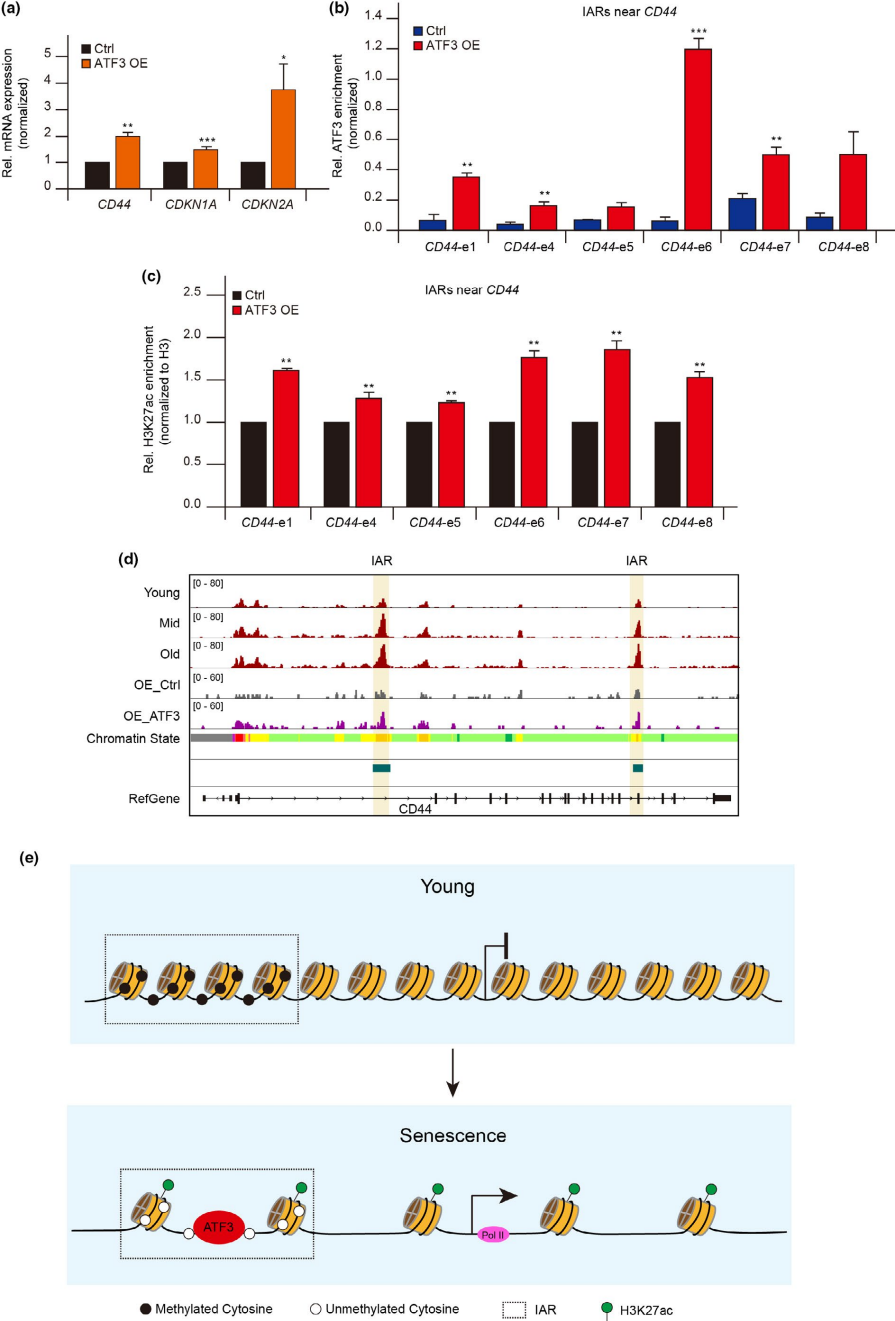
ATF3 is responsible for accessibility reprogramming of IARs. (a) mRNA levels of *CD44*, *CDKN1A*, and *CDKN2A* in HUVECs after ATF3 overexpression. The cycle threshold (Ct) of *ATF3* and *CDKN2A* was normalized with *ACTB*. (b) ChIP‐qPCR analysis of ATF3 binding at IARs in or near *CD44* after ATF3 overexpression. The *y*‐axis represents the percentage of ATF3 enrichment relative to input. (c) ChIP‐qPCR showing the H3K27ac signals at IARs after ATF3 overexpression in HUVECs. The *y*‐axis represents relative H3K27ac levels in ATF3‐overexpressed HUVECs and in control cells. The relative H3K27ac levels were normalized to H3. The error bars represent the s.d. obtained from triplicate independent experiments. Two‐tailed, unpaired Student's *t* tests were performed. *p < 0.05, **p < 0.01, and ***p < 0.001. (d) Snapshot showing the ATAC‐seq peaks near *CD44* in ATF3‐overexpressed HUVECs. Vertical shadows represent the IARs. (e) Proposed model showing ATF3 promoting senescence by remodeling the chromatin accessibility of IARs, which is affected by DNA methylation

Additionally, the enrichment of ATF3 in IARs was increased during HUVEC senescence (Figure S11). After *ATF3* overexpression, the ATF3 binding at IARs were enhanced (Figure 6b and Figure S12) and the expression of the genes surrounding IARs was increased (Figure 6a and Figure S13a,b). Meantime, the H3K27ac, H3K4me1, and ATAC‐seq signals at IARs were also improved (Figure 6c,d, Figure S13c–h, and Figure S14). These results demonstrated that ATF3 binds to IARs to open the chromatin, and further activate the neighboring gene expression. In summary, the AP‐1 family, especially *ATF3*, is involved in the establishment of a senescence program that regulates the accessibility in IARs, thus further reconstructing the senescence‐related gene expression profile (Figure 6e).

## 3 DISCUSSION

We found that the gene expression profile in senescent cells is predetermined by accessible chromatin rearrangement. The senescence‐specific chromatin regions, IARs and DARs, have distinct functions in senescence, with IARs involved in senescence regulation and DARs related to declining cell function caused by senescence and involving weak enhancers and promoters. But most importantly, we showed that the gradual increase in IAR accessibility during senescence is driven primarily by AP‐1 transcription factors, especially ATF3. The reshaping of chromatin accessibility by AP‐1 transcription factors, such as ATF3, is affected by DNA methylation at their binding sites. In general, ATF3 significantly affects reconstruction of the gene expression program, thus further promoting senescence that is mediated by regulatory elements such as enhancers in IARs. Therefore, ATF3 is paramount in the senescence regulatory network and may be an effective target for senescence intervention.

Senescence fate depends on an epigenetic pattern, a systemic state in which chromatin remodeling, histone modifications, and DNA methylation cooperatively decide the cell future. But the understanding about the interactions and priorities of those processes is still incomplete. We systematically analyzed chromatin accessibility remodeling in different stages of senescence using ATAC‐seq technology. Our results demonstrated that senescence adheres to a program mainly scheduled by chromatin architecture. The consistent trajectory between chromatin accessibility and gene expression during senescence shows a possible regulatory direction in which senescence‐specific gene expression profiles are determined by chromatin rearrangement patterns (Figure 1f and Figure 3a,b). Among them, *FOXA1*, *PLAT*, and other genes are up‐regulated, whereas *ZNF423*, *PHGDH*, and other genes are down‐regulated in multiple senescence systems (Galanos et al., 2016; Hernandez‐Segura et al., 2017; Komseli et al., 2018), as are consistent with our results. These genes are located in or near the regions of IARs or DARs, respectively. For a long time, the workings of senescence were elusive, but eventually increasing evidence pointed to chromatin architecture remodeling as a possible unifying theory that could describe serial events in senescence. As a holistic process, senescence involves multiple pathways, each related to many genes. Changing the expression of multiple senescence‐related genes at the same time may be an effective senescence intervention strategy. Ocampo et al. (2016) ameliorated senescence and the aging phenotype by executing short‐term cyclic expression of *Oct4*, *Sox2*, *Klf4*, and *c*‐*Myc* in mice. Sarkar et al. (2020) transiently expressed *OCT4*, *SOX2*, *KLF4*, c‐MYC, *LIN28*, and *NANOG* in human cells, partially reversing cellular senescence and restoring aged tissue. Those actions removed the senescence program by enacting specific changes to the chromatin landscape that rearranges the gene expression profile. Our whole‐genome chromatin accessibility and gene expression landscape provide a global perspective to senescence, thus enabling the ability to find intervention targets for senescence and aging. Our research found that the accessible chromatin significantly rearranged is not near TSSs and that most of it lies in intronic and intergenic regions where distal regulatory elements are plentiful (Figures 1g and 2b, Figures S5b and S7a). Perhaps many senescence‐associated regulatory elements are hidden in distal intergenic regions rather than in proximal regions.

Most notably, we identified senescence‐specific chromatin regions, IARs, and found that the drivers facilitating those regions are AP‐1 transcription factors that function as pioneer factors. Meanwhile, we noticed that IARs are mostly comprised of heterochromatin (Figure S7), which was transformed to open chromatin by gaining accessibility, thus indicating a potentially special heterochromatin contribution to senescence. Meanwhile, decreased DNA methylation levels at AP‐1 binding sites promote its enrichment in IARs, which successfully reshapes the accessibility pattern of adjacent chromatin (Figures 4 and 6b). Our results indicate that the binding of AP‐1 is sensitive to DNA methylation. Martinez‐Zamudio et al. (2020) reported that AP‐1 transcription factors regulate the senescence program of oncogene‐induced senescence in WI38 fibroblasts, a result that parallels our work replicating senescence in HUVECs. Even though those researchers used different cell lines and different types of senescence than we did, we both found that the AP‐1 transcription factors family can regulate senescence fate by remodeling gene expression profiles and that perturbation of AP‐1 transcription factors’ expression can reverse the senescence program. However, they identified c‐JUN as the core AP‐1 transcription factor that regulates senescence, unlike our finding that ATF3 has that role. This shows that while AP‐1 transcription factors are drivers in senescence, exactly which of those factors are involved and what their possible functional combinations can vary given the cells and senescence types. These gaps in knowledge must be filled by exploring the functions of the AP‐1 family genes in senescence to discover more specific targets for interventional strategies for aging.

Recent studies have implied that AP‐1 could drive breast cancer and oncogene‐induced senescence by regulating the accessibilities of specific promoters and enhancers (Han et al., 2018; Vierbuchen et al., 2017). A functional CRISPR screen identified an AP‐1‐associated enhancer that modulates oncogene‐induced senescence by regulating *FOXF1* (Han et al., 2018). We knocked down other members of AP‐1 family (*FOSL1*, *FOSL2*, and c‐*JUN*), and we found that their defection delayed senescence phenotype (Figure S15). In summary, we proposed that AP‐1 is a significant prosenescence regulator. Further research needs to flesh out the detailed mechanisms, such as the interaction network and action time, of chromatin accessibility redistribution regulation by AP‐1 during senescence. Also, well‐elucidated chromatin landscapes in multiple senescence systems are needed.

In all, we presented a sequentially dynamic landscape orchestrating chromatin accessibility and gene expression during the entire culture process for human primary cells in vitro and defined IARs and DARs as two kinds of senescence‐specific chromatin regions. Also, DNA methylation possibly affects AP‐1 transcription factor binding to the regulatory elements in IARs. We showed that AP‐1 transcription factors, especially ATF3, promote senescence by remodeling chromatin accessibility in IARs. Accordingly, our research provides a target to aim for in the exploration of senescence and aging.

## 4 EXPERIMENTAL PROCEDURES

### 4.1 Cell culture

We purchased primary HUVECs from AllCells Biotech Shanghai Co., Ltd., and cultured them in endothelial cell medium (ScienCell Research Laboratories, Inc.). HEK293 T cells, purchased from American Type Culture Collection (Manassas, VA, USA), were cultured in Gibco Dulbecco's modified Eagle's medium (DMEM) (Fisher Scientific), supplemented with 10% fetal bovine serum (FBS, Fisher Scientific). All cells were maintained at 37°C in a humidified incubator with 5% CO_2_. When HUVECs attained 80%–90% density, usually in 2 or 3 days with 0.5 × 10^6^ start‐up cells, they were passaged in a 10‐cm dish coated with 0.25% gelatin and then cultured until they ceased proliferation.

### 4.2 ATAC‐seq

We scraped out the freshly cultured HUVECs to cold 1× phosphate‐buffered saline (PBS) that were at various points in the senescence time course. The samples were centrifuged with 500 g, 4°C for 5 min. The cell pellet was resuspended with moderately cold lysis buffer (400 μl lysis buffer for 1.0 × 10^6^ cells), then split out 7.0 × 10^4^ lysed cells to a sterile PCR tube for the subsequent transposition reaction and library construction using the TruePrep DNA Library Prep Kit V2 (Vazyme Biotech Co., Ltd). 5× TTBL, TTE Mix V50, and ddH_2_O were added to lysate precipitation according to the instructions. The samples were heated at 37°C for 30 min to perform the transposition reaction. The transposed DNA was purified using the MinElute PCR Purification Kit #28004 (Qiagen) according to the kit instructions. DNA libraries were produced after eight cycles of PCR amplification using the TruePrep library prep kit. After purification, the paired‐end sequencing was performed on a HiSeq PE150 sequencer (Illumina) at Novogene Biotech Co., Ltd. ×10^3^.

### 4.3 RNA‐seq

TRIzol reagent (Invitrogen) was used according to package instructions to extract total RNA. Subsequently, we sent the RNA to Novogene for subsequent cDNA library construction and Illumina sequencing with paired‐end reads on a HiSeq PE150.

### 4.4 Senescence‐associated β‐galactosidase (SA‐β‐gal) staining

Before the SA‐β‐gal staining assay, about 0.6 × 10^5^ cells were seeded into each well of 6‐well plates. We used a Senescence Cells Histochemical Staining Kit (Sigma) as follows. Briefly, the adherent cells were washed twice with 1× PBS after aspirating the media. Cells were fixed with 1× fixation buffer and incubated for 7 min at room temperature. After rinsing the cells three times with 1× PBS, we added prepared staining mixture to all experimental wells. Next, the fixed cells were incubated overnight at 37°C, and then, images acquired using a DMI 6000B inverted microscope with 10 × 10 magnification (Leica) were analyzed using the ImageJ software (NIH) to calculate the percentage of senescing cells based on SA‐β‐gal signals.

### 4.5 Immunofluorescence

About 0.5 × 10^6^ cells were seeded in 10‐cm dishes with sterile coverslips and cultured about 48 h. The cells on the coverslips were fixed for 10 min at room temperature with 4% paraformaldehyde after washing twice with cold 1× PBS. Then, the cells were blocked for 30 min with 1% bovine serum albumin at room temperature, washed with 1× PBS, and then incubated overnight at 4°C with 80 μl primary antibody, anti‐Ki67(ab15580; Abcam), diluted 1:500 with PBS Tween‐20 (PBST). Later, the cells were washed four times with 0.1% PBST and incubated for 2 h at room temperature with 80 μl secondary antibody, Alexa Fluor 594 Donkey anti‐rabbit IgG, diluted 1:500 with PBST (Life Technologies). After washing with PBST, the cells were incubated for 3 min with 1 ng/μl DAPI fluorescent stain at room temperature, and then, the coverslips were mounted and sealed with 10 μl Fluoromount‐G. One hour later, we acquired images using a fluorescent microscope and used ImageJ software to analyze the immunofluorescence signals.

### 4.6 qPCR methods

#### 4.6.1 ChIP‐qPCR

The cells used for the ChIP assay were cultured to about 80% confluence, and then, 1% formaldehyde and 0.125 M glycine were added to the media to initiate and terminate crosslinking, respectively. The scraped cells were placed in a 1.5‐ml DNA LoBind Tube (Eppendorf) and centrifuged at 2500 *g* and 4°C for 5 min. The pellet was then resuspended with nuclei lysis buffer (50 mM Tris‐Cl, 10 mM EDTA, 1% SDS, and a protease inhibitor cocktail) and the released chromatin was sonicated using a Bioruptor ultrasonicator (Diagenode) before being diluted with IP dilution buffer (20 mM Tris‐Cl, 2 mM EDTA, 150 mM NaCl, 1% Triton X‐100 and a protease inhibitor cocktail). The samples were incubated with specific antibodies overnight at 4°C. The antibodies were anti‐H3K4me1 (ab8895, Abcam), anti‐H3K27ac (ab4729, Abcam), and anti‐H3 (ab1791, Abcam). And the chromatin–antibody complexes were captured by protein A/G beads. We used qPCR to assay the immunoprecipitated DNA. The primers used in this analysis are in Table S1.

#### 4.6.2 RT‐qPCR

We used TRIzol reagent according to the included instructions to extract total RNA from cultured cells at 80% confluence. Then, the RNA resolved in DEPC‐treated water was reverse‐transcribed to cDNA using All‐in‐One Supermix (TransGen Biotech Co., Ltd.) according to the product instructions. We used a LightCycler^®^ 96 qPCR Instrument (Roche) to analyze cDNA levels with specific primers and normalized the results to β‐actin. All qPCR‐related primers are available in Table S2.

### 4.7 Knockdown and overexpression

For ATF3 overexpression, the pSIN‐3×flag‐ATF3 vector, which was derived from the pSIN‐EF2‐Nanog‐Puro Plasmid (Addgene), was packaged by lentivirus and then transduced to HUVECs. For ATF3 knockdown, we purchased small interfering RNA (siRNA) of *ATF3*, *FOSL1*, *FOSL2*, and *c*‐*JUN* from GenePharma Co., Ltd. siRNA was transfected into HUVECs using Lipo3000 (Thermo Fisher Scientific China Co., Ltd.) according to manual. The knock‐down experiments with siRNA oligos were performed using PD14 HUVECs.

### 4.8 Lentiviral production and viral transduction

HEK293 T cells were seeded so that the cell density would be 80% confluent at the time of transient transfection. The target constructs, psPAX and pMD2.G, were cotransfected using calcium phosphate. The media were replaced with DMEM supplemented with 30% complement‐inactivated FBS after 18 h of transfection, and viral supernatants were collected after 48 h of transfection. When HUVEC cell density reached 60% confluence, the cells were infected by the packaged viruses with 5 μg/ml polybrene (M&C Gene Technology), and 36–48 h after infection, transduced cells were selected with 0.5 μg/ml puromycin (M&C Gene Technology Ltd).

### 4.9 Western blotting

Proteins were extracted using TRIzol reagent (Invitrogen) and resolved in 1% SDS. After SDS‐PAGE, the samples were transferred to nitrocellulose membranes and incubated for 30 min at room temperature with 5% skimmed milk. The membranes were incubated overnight at 4°C with specific primary antibodies: anti‐Lamin B1 (1:1000, 12987‐1‐AP, Proteintech), anti‐β‐actin (1:2000, sc‐47778, Santa Cruz Biotechnology), anti‐p16 (1:500, ab108349, Abcam), anti‐ATF3 (1:500, ab207434, Abcam), and anti‐Tublin (1:1000, T6199, Sigma). After washing three times with PBST (1× PBS and 0.1% Tween‐20), the membranes were incubated at room temperature for 2 h with IRDye 800CW Goat/Donkey anti‐Mouse/Rabbit secondary antibodies (1:10,000, 926‐32210; LI‐COR Biosciences). We acquired blot information using the Odyssey Infrared Imaging System (LI‐COR).

### 4.10 Data analysis

#### 4.10.1 RNA‐seq data

Sequencing adapters were removed from the raw RNA‐seq reads, and the trimmed reads were then mapped to the hg19 human genome using TopHat v2.1.0 (https://ccb.jhu.edu/software/tophat/manual.shtml). To quantify gene expression, we counted the sequencing reads within each gene and normalized the counts using edgeR (Robinson et al., 2009).

#### 4.10.2 ATAC‐seq data

We removed sequencing adaptors from the raw ATAC‐seq reads, and the clean data were mapped to the human reference genome using Bowtie2 with the following parameters: “‐L 25 ‐X 2000 ‐t ‐q ‐N 1 –no‐mixed –no‐discordant.” PCR duplicates were removed using Picard, and then, MACS, using parameters “‐F BAM ‐g hs ‐w –nolambda ‐‐nomodel,” was used to call significant peaks.

#### 4.10.3 Identification of IARs and DARs

After we had merged all peaks from each sample into a single file, we calculated the ATAC‐seq reads in each sample's merged peak file. To identify the different accessibility peaks, we used edgeR in R to normalize the data and call the IARs and DARs. The criteria for DEGs and IARs/DARs are used edgeR package (Robinson et al., 2009). The expression/accessible matrix was normalized by TMM method, and then, differentially expressed genes/accessible regions were defined by comparing old cells with young cells, and the midlife cells were not used for the comparison.

#### 4.10.4 Genomic Region Enrichment of Annotation Tool (GREAT) analysis

To perform gene ontology enrichment analysis of IARs and DARs, we uploaded the IAR and DAR genomic regions in bed format to the GREAT server (http://great.stanford.edu/public/html/).

#### 4.10.5 Motif enrichment analysis

We used the findMotifsGenome.pl function in the HOMER toolkit (http://homer.ucsd.edu/homer/motif/) to find the motifs in the IARs and DARs and then used CentriMo in MEME Suite (http://meme‐suite.org/doc/centrimo.html) to search the significant motifs enriched in the IARs and DARs across the human core motif database.

## CONFLICT OF INTEREST

The authors declare no competing interests.

## Supporting information

Supplementary Material

Table S4

Table S5

## Data Availability

The ATAC‐seq sequencing data of this study are available at GEO under accession number: GSE157867.
