# Supplementary material for: ATF3 drives senescence by reconstructing accessible chromatin profiles
Source: Aging Cell. 2021 Feb 4;20(3):e13315. doi: 10.1111/acel.13315 (PMC7963335; doi:10.1111/acel.13315)
Supplement: Supplementary file 1 — Supplementary Material [file ACEL-20-e13315-s002.docx]

**SUPPORTING INFORMATION**

**Supplementary Figures**


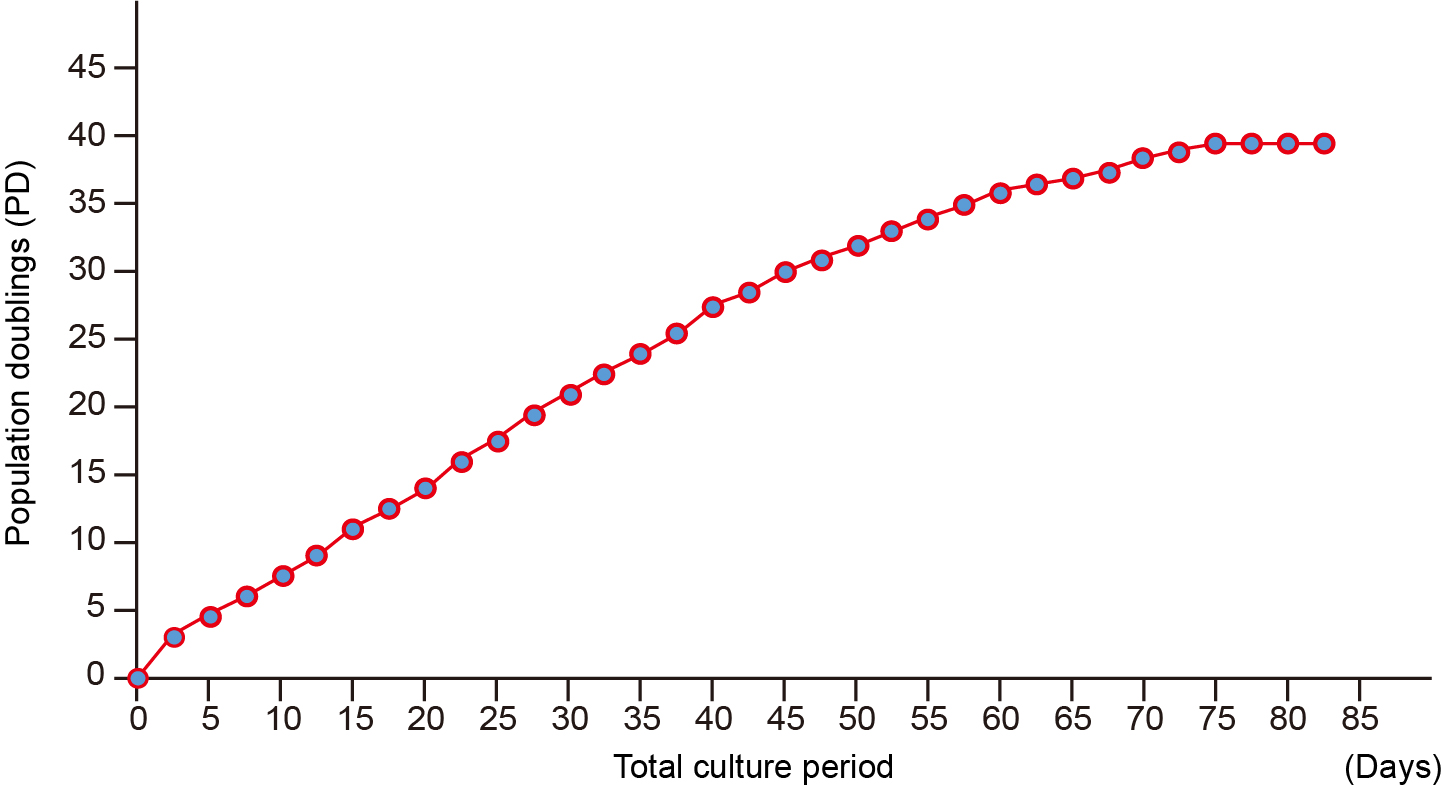
**Figure S1.** Growth curve of human umbilical vein endothelial cells (HUVECs) cultured *in vitro*. Each culture was initially inoculated with 0.5 × 10^6^ cells and allowed to grow to 80% to 90% confluence for 1 passage before the cells were counted. PD = log (N/N0) + S, where N represents inoculant amount in a specific period, N0 represents the initial inoculation amount, and S represents the initial cell proliferation generation


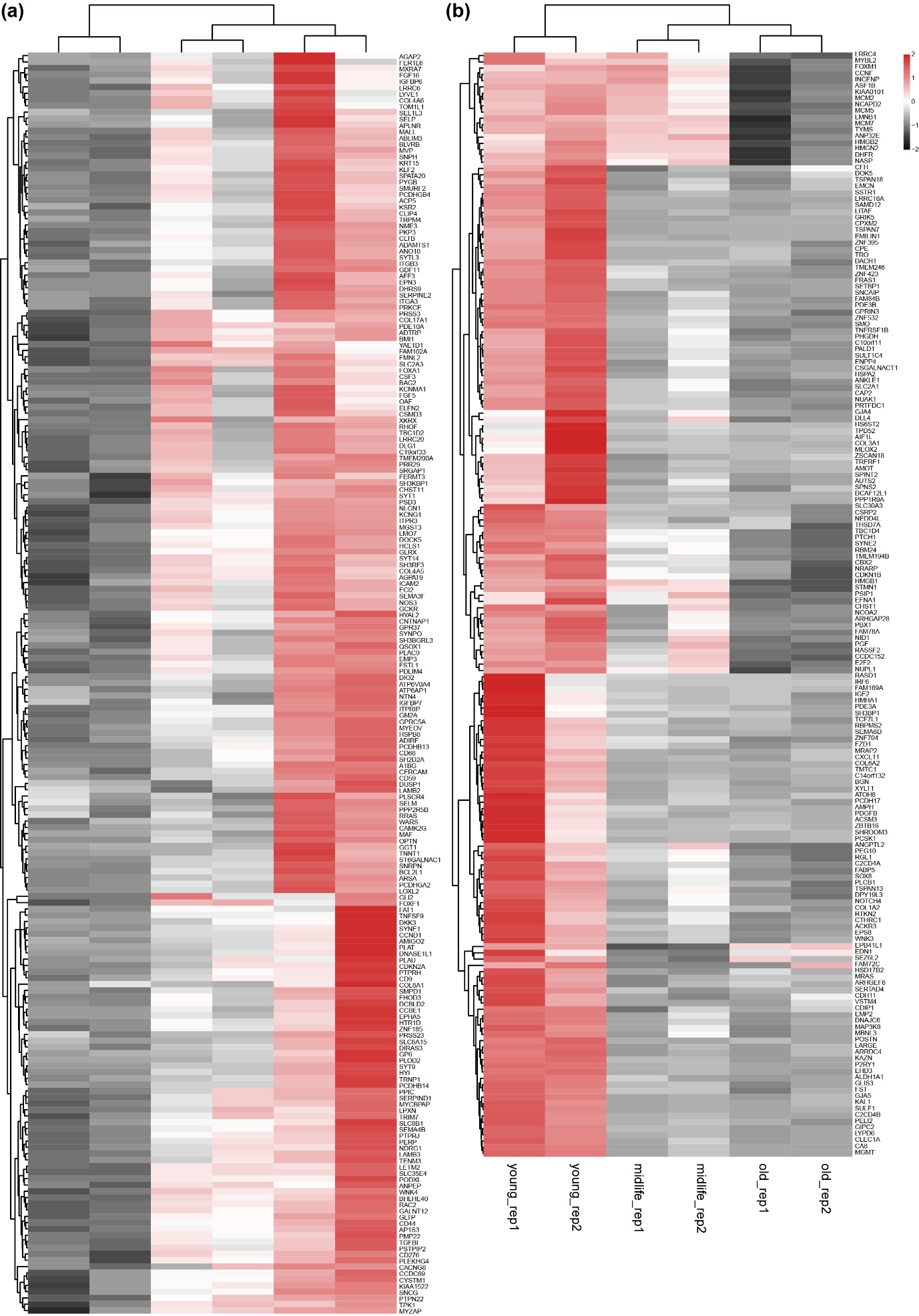


**Figure S2.** Heatmaps of RNA-seq results showing hierarchically clustered gene expressions in HUVEC senescence. (a) Significantly upregulated genes. (b) Significantly downregulated genes. Young, population doubling 8 cells (PD8); Mid, population doubling 24 cells (PD24); Old, population doubling 36 cells (PD36). Two biological replicates were performed in each category (rep 1 and 2).


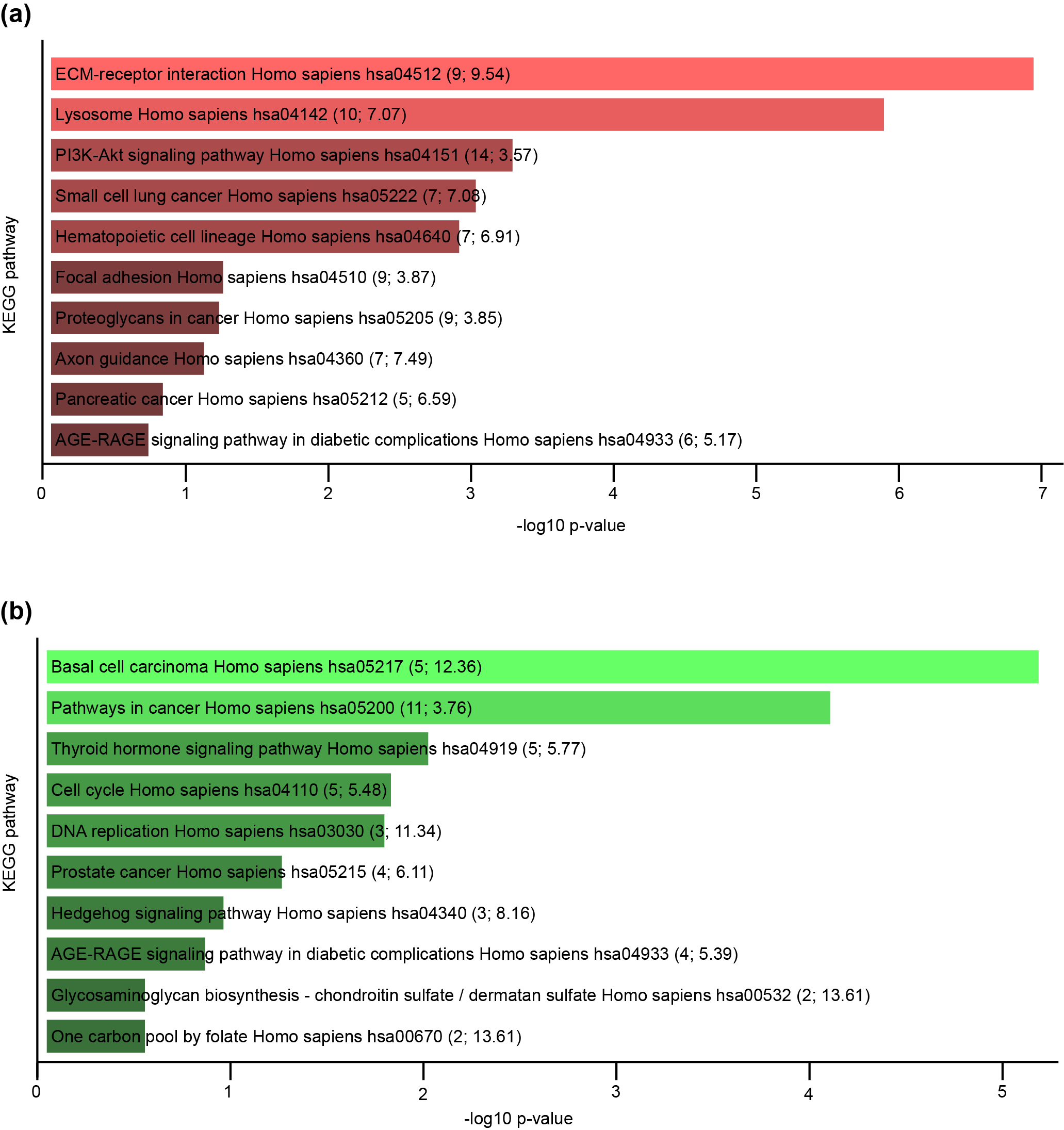
**Figure S3.** KEGG pathway enrichments analysis of Up/Down regulated genes during HUVECs senescence. (a) Upregulated signaling pathways (b) Downregulated signaling pathways.

**
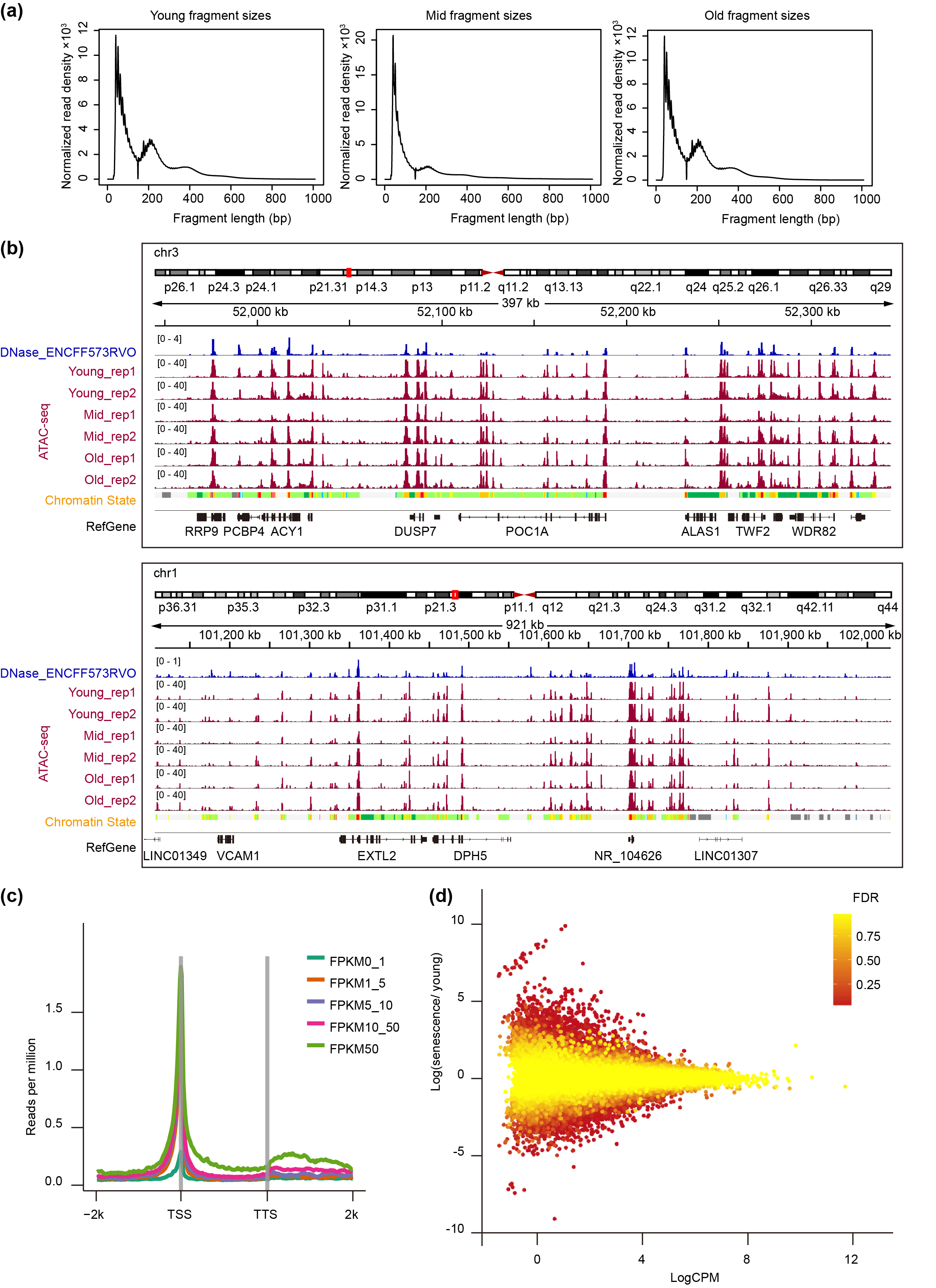
Figure S4.** ATAC-seq data quality analysis. (a) The distribution of fragment insertion sizes of ATAC-seq libraries. (b) Comparison of our ATAC-seq and the ENCODE DNase-seq data sets showed marked agreement. (c) Average ATAC-seq signals around gene bodies. Genes were divided into 5 fragments per kb of transcript (FBKM) classes according to increasing expression levels: 1 to 50 reads per million. TSS, transcription start sites; TTS, transcription termination site. (d) Scatter plot of ATAC-seq signals showing the importance of accessibility change. See Figure S2 for senescence class definitions. CPM, count per million reads. FDR, false discovery rate.

**
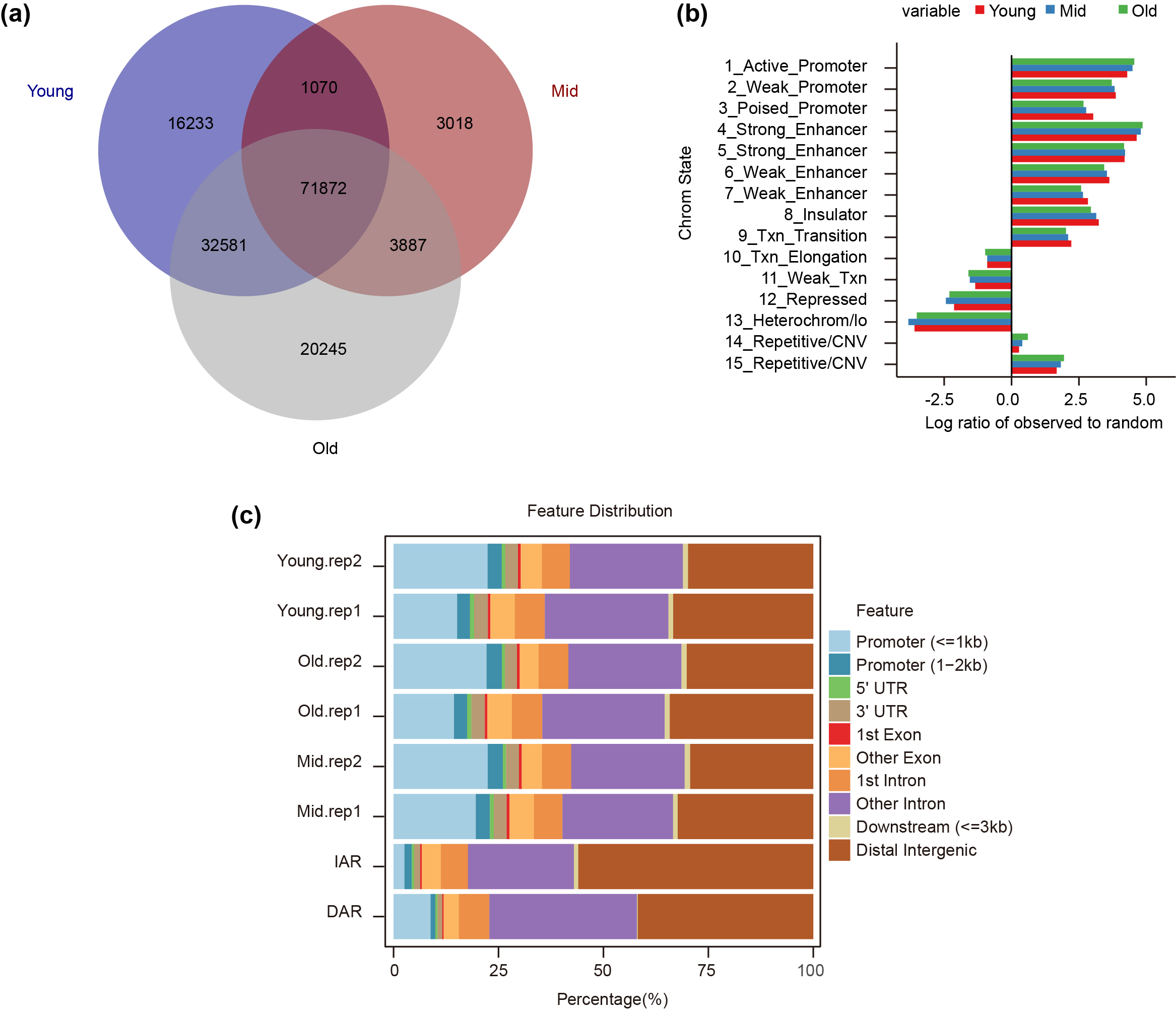
Figure S5.** Characteristics of ATAC-seq signal distributions among different senescence stages (defined in Figure S2). (a) Venn diagram showing the overlapping ATAC-seq signals among the 3 senescence stages. Each number represents the ATAC-seq peaks. (b) Relationships of chromatin states for significantly changed regions of chromatin accessibility during senescence stages. Txn, transcribed region; CNV, copy number variation; lo, low signal. (c) Genomic feature distributions of changed accessible chromatin regions during senescence stages. Two independently biological replicates were performed in each category (rep 1 and 2). UTR, untranslated region.


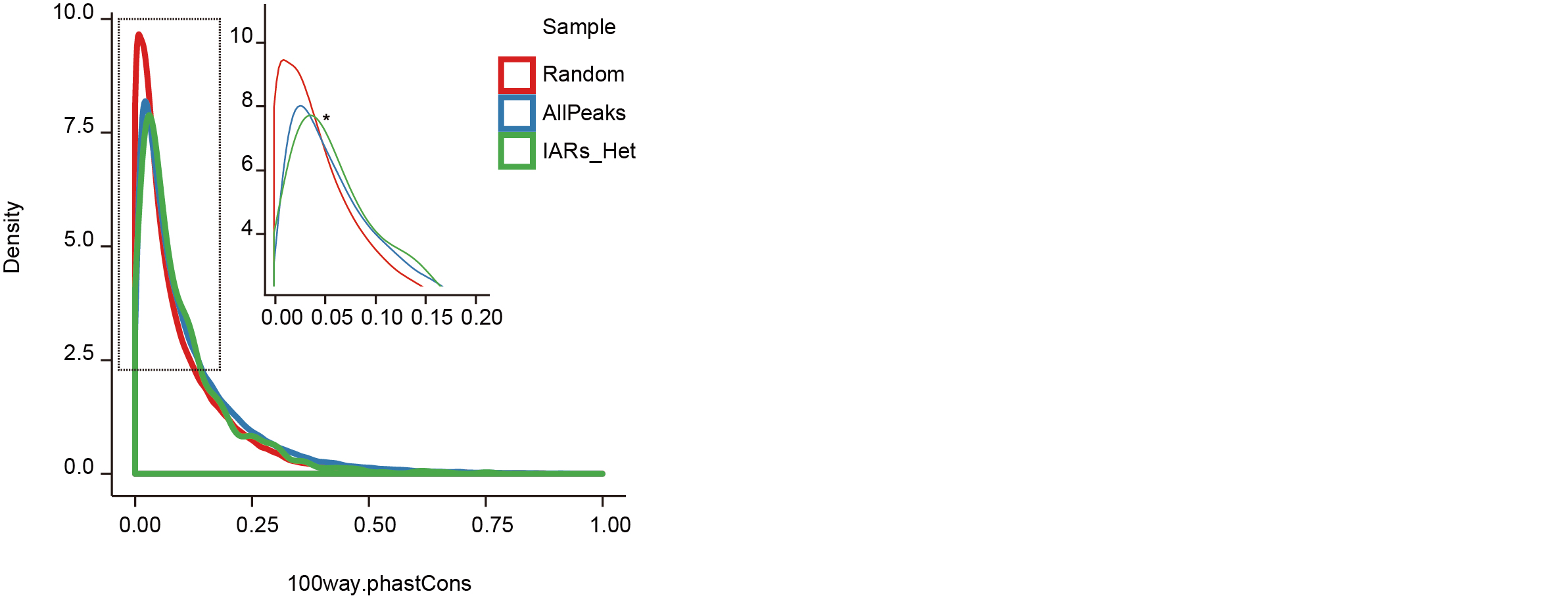
**Figure S6.** Conservation scores distribution of IAR, all ATAC-seq peaks, and random regions. The inset graph shows the area within the boxed section of the larger graph. Two-tailed, unpaired Student’s *t*-tests were performed. **P* < 0.05.


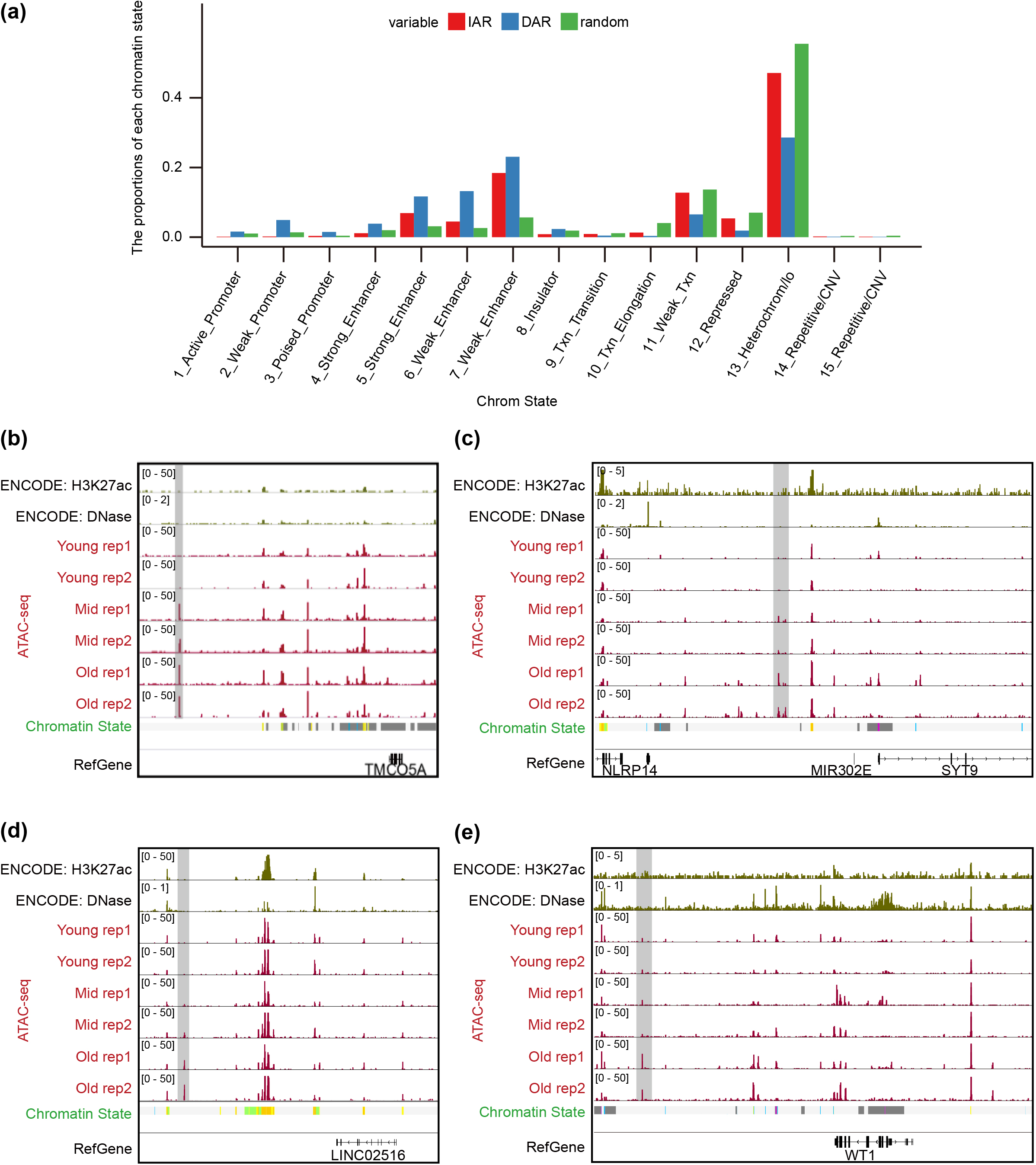
**Figure S7.** Heterochromatin contributes to senescence-related chromatin regions with increased accessibility (IARs). (a) Percentage of each chromatin state in IARs and DARs. The “random” means random regions, which is selected as control for displaying the distribution of genomic background. (b-e) Snapshots showing the ATAC-seq signals near *TMCO5A* (b), *SYT9* (c), *LINC02516* (d), and *WT1* (e). Public H3K27ac and DNase-seq signals from primary HUVECs are also shown at the top. Chromatin states were obtained from ENCODE (light yellow = weak/poised enhancer, dark yellow = strong enhancer, green = transcribed region, blue = insulator, gray = heterochromatin). The gray vertical bar highlights the IARs in heterochromatin regions.


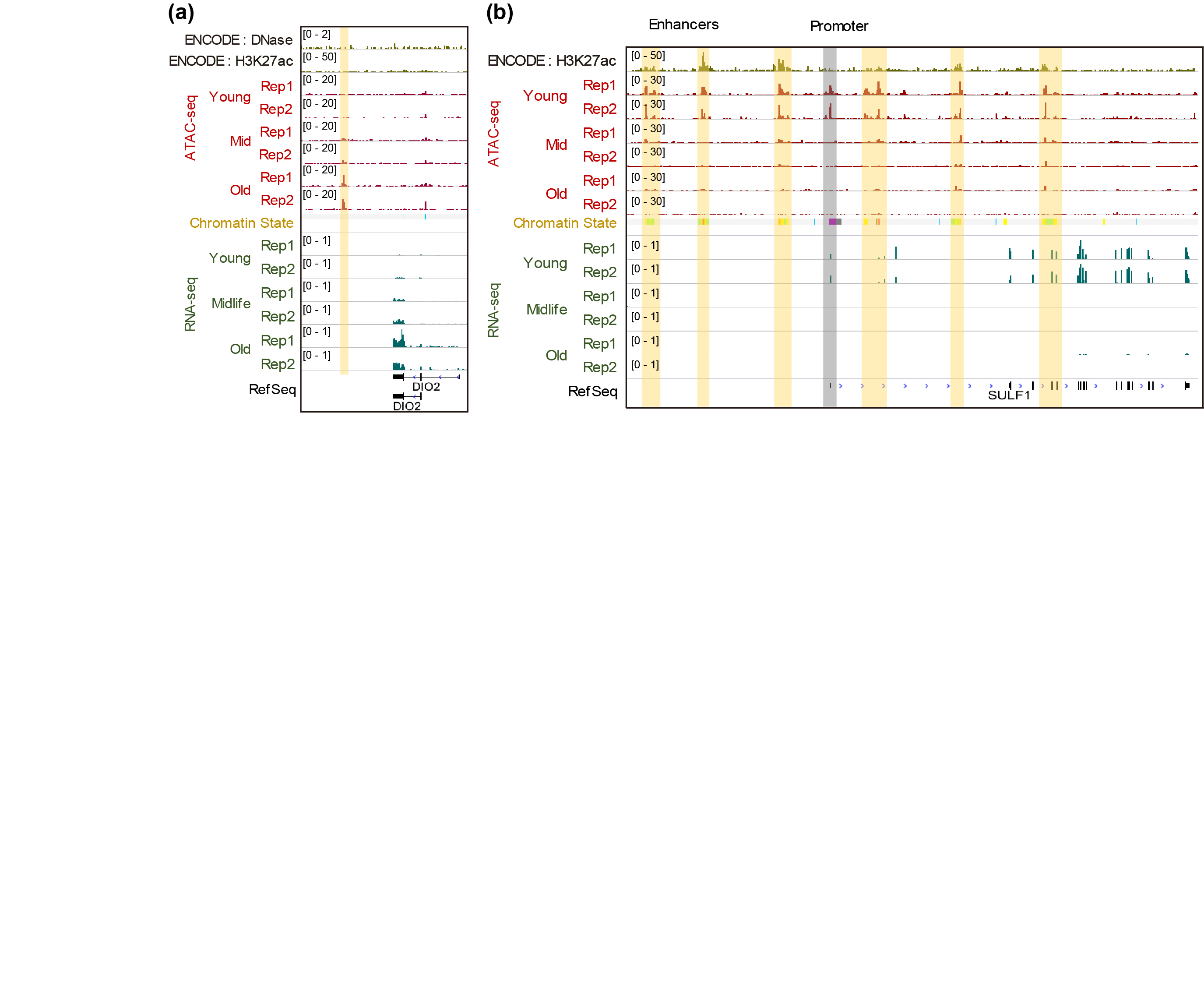
**Figure S8.** Examples of IAR and DAR. (a, b) Snapshots showing the corresponding changes in ATAC-seq and RNA-seq peaks in or near *DIO2* (a) and *SULF1* (b) during senescence. The vertical yellow boxes represent IARs and DARs in enhancers, the gray vertical box represent DAR in promoter. Two biological replicates were performed in each category (rep 1 and 2). See Figure S2 for senescence stage definitions and Figure S7 for chromatin state information

**
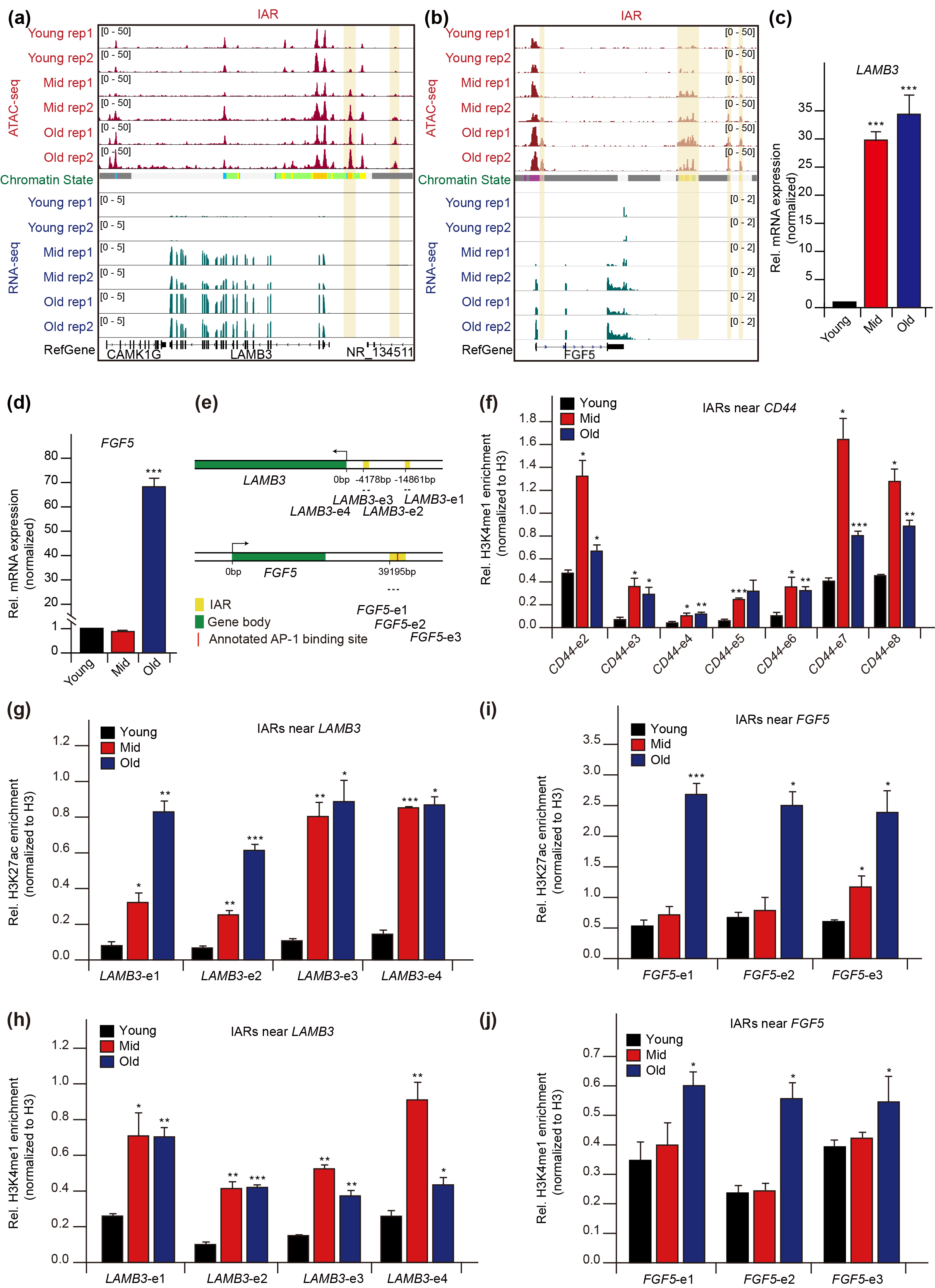
Figure S9.** Chromatin accessibility of IARs in HUVEC senescence. (a, b) ATAC-seq and RNA-seq signals near *LAMB3* (a) and *FGF5* (b). IARs are highlighted with vertical yellow boxes. Two biological replicates were performed in each category (rep 1 and 2). See Figure S7 for chromatin state information. (c, d) RT-qPCR results showing mRNA levels of *LAMB3* (c) and *FGF5* (d) during HUVECs senescence. The cycle threshold (Ct) of *LAMB3* and *FGF5* was normalized with *ACTB*. (e) Locations of ChIP-qPCR primers relative to *LAMB3* and *FGF5*. (f-j) ChIP-qPCR analysis of H3K4me1 at IARs in or near *CD44* (f), H3K27ac at IARs near *LAMB3* (g), H3K4me1 at IARs near *LAMB3* (h), H3K27ac at IARs near *FGF5* (i), and H3K4me1 at IARs near *FGF5* (j). See Figure S2 for senescence stage definitions. The y-axis represents the percentage of H3K27ac or H3K4me1 signals relative to input which were normalized to H3. The error bars represent the s.d. obtained from triplicate independent experiments. Two-tailed, unpaired Student’s *t*-tests were performed. **P* < 0.05, ***P* < 0.01, ****P* < 0.001.

**
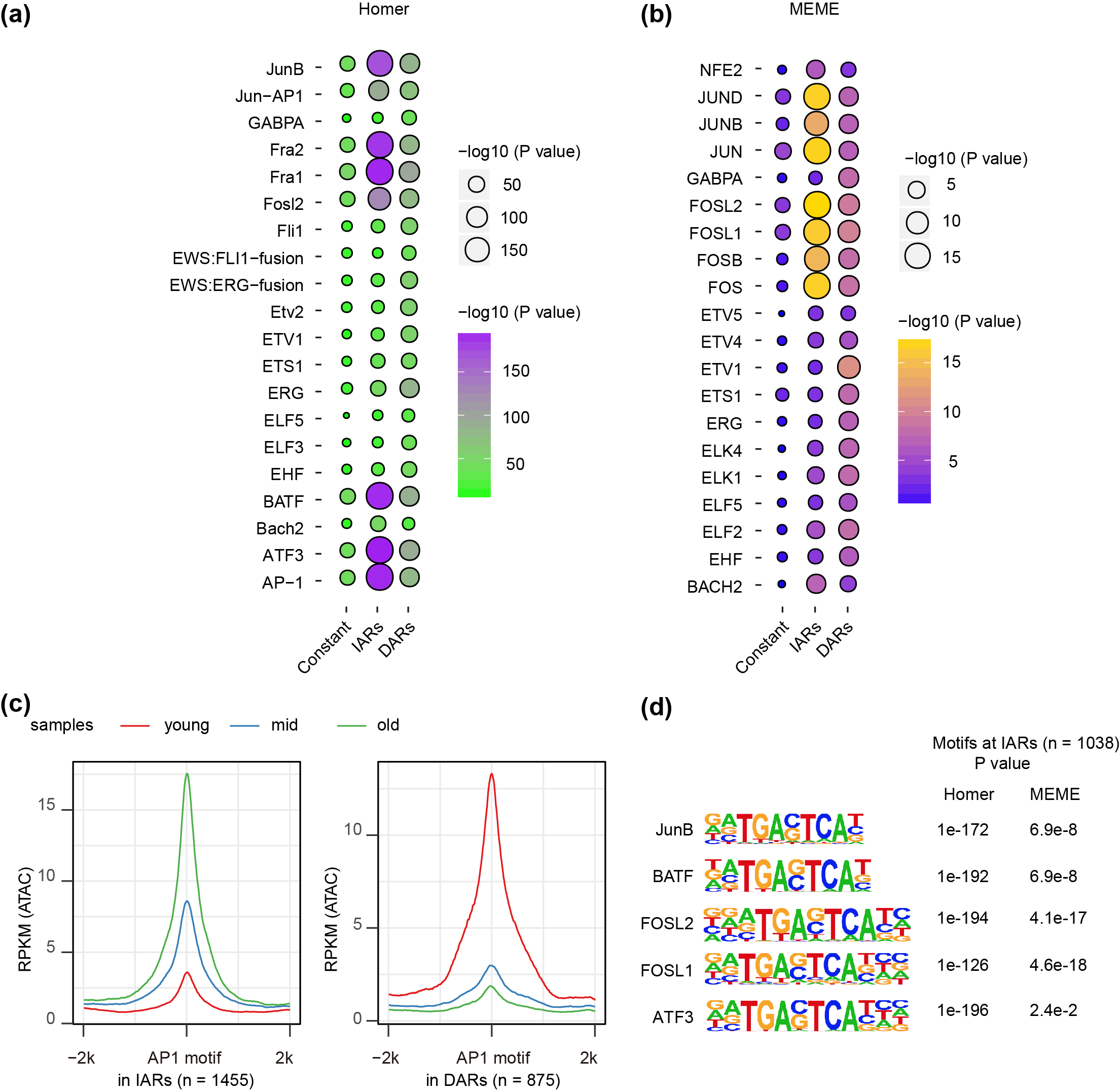
Figure S10.** AP-1 transcription factors identified in senescence. (a, b) Motif analysis results using Homer and MEME algorithms for transcription factors at IARs and DARs. (c) Comparisons of ATAC-seq signals of AP-1 motif at IARs and DARs. See Figure S2 for senescence stage definitions. RPKM, reads per kilobase million. (d) Comparisons of selected AP-1 family members’ motif enrichment analyses in IARs using Homer and MEME algorithms. The results show variations in the levels of enrichment for each transcription factor.


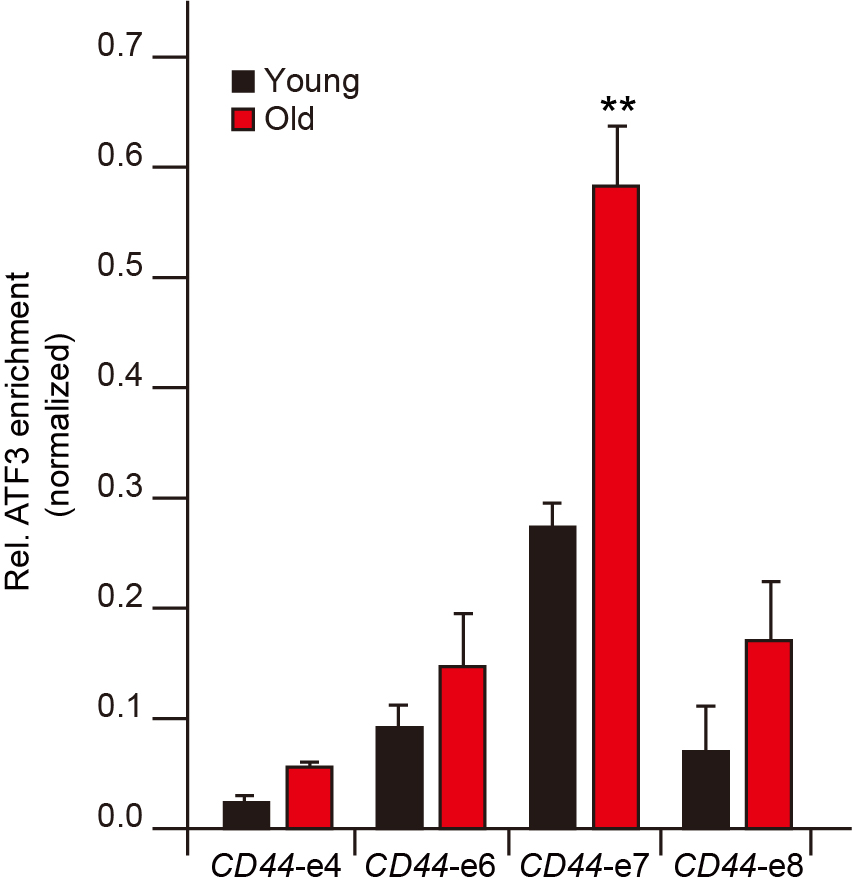
**Figure S11.** The enrichment of ATF3 at IARs during HUVECs senescence. ChIP-qPCR assay for ATF3 in young and old HUVECs showing the changes of ATF3 binding at IARs during HUVECs senescence. The y-axis represents the percentage of ATF3 enrichment relative to input. The error bars represent the s.d. obtained from triplicate independent experiments. Two-tailed, unpaired Student’s t-tests were performed. **P < 0.01.


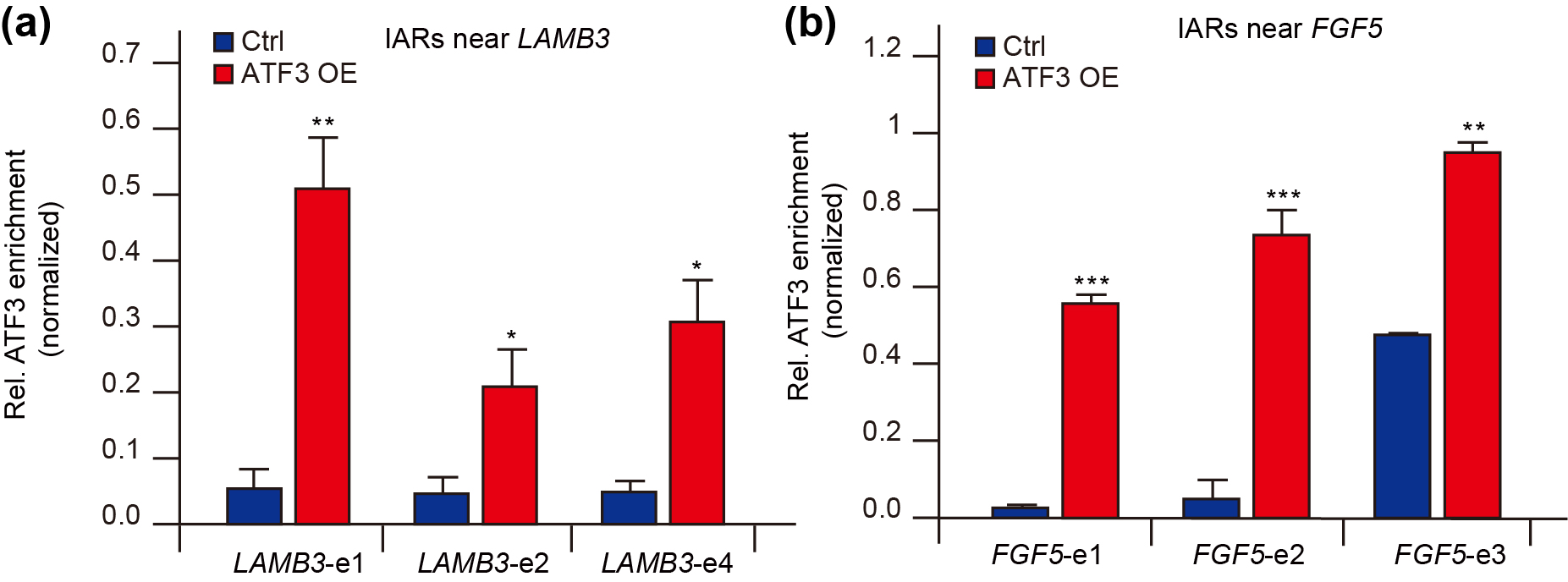
**Figure S12.** The enrichment of ATF3 at IARs after ATF3 overexpression in HUVECs. (a, b) ChIP-qPCR results showing the enrichment of ATF3 at IARs near *LAMB3* (a) or *FGF5* (b) in ATF3-overexpressed or control HUVECs. The y-axis represents the percentage of ATF3 enrichment relative to input. The error bars represent the s.d. obtained from triplicate independent experiments. Two-tailed, unpaired Student’s t-tests were performed. **P < 0.01, ***P* < 0.01, ****P* < 0.001.

**
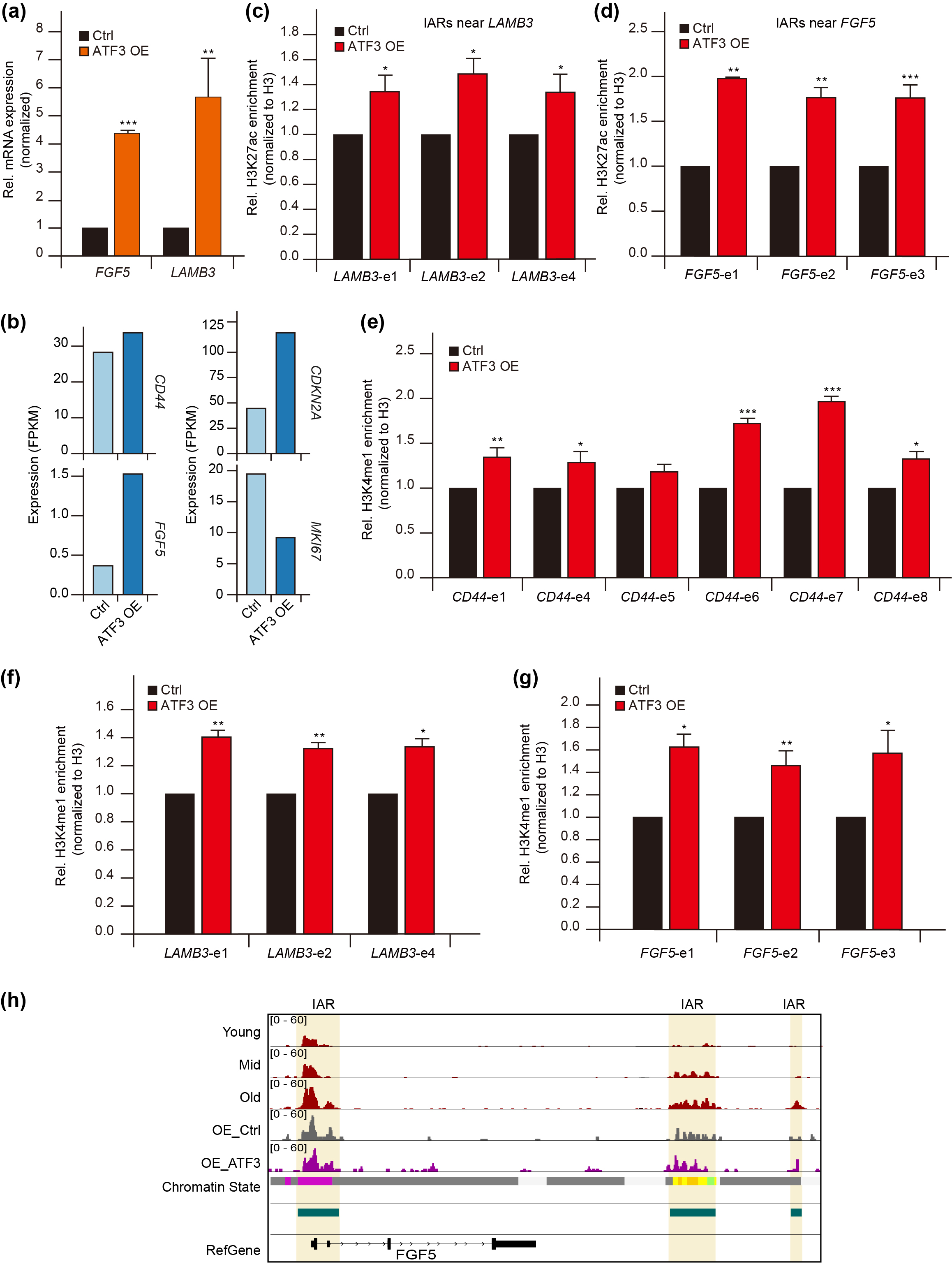
Figure S13.** Chromatin accessibility of IARs after ATF3 overexpression in HUVECs. (a) RT-qPCR results showing mRNA levels of *FGF5* and *LAMB3* after ATF3 overexpression. (b) RNA-seq results showing mRNA levels of *CD44* and *FGF5* after ATF3 overexpression. FPKM, fragments per kilobase million. (c, d) H3K27ac ChIP-qPCR assays for the IARs near *LAMB3* (c), and *FGF5* (d). The y-axis represents the percentage of H3K27ac signals relative to input which were normalized to H3. (e-g) H3K4me1 ChIP-qPCR assays for the IARs near *CD44* (e), *LAMB3* (f) and *FGF5* (g). The y-axis represents the percentage of H3K4me1 signals relative to input which were normalized to H3. See ChIP-qPCR primers location in Figure3 and Figure S9. The error bars represent the s.d. obtained from triplicate independent experiments. Two-tailed, unpaired Student’s *t*-tests were performed. **P* < 0.05, ***P* < 0.01, ****P* < 0.001. (h) Snapshot showing the ATAC-seq peaks at IARs near FGF5 after ATF3 overexpression.

**
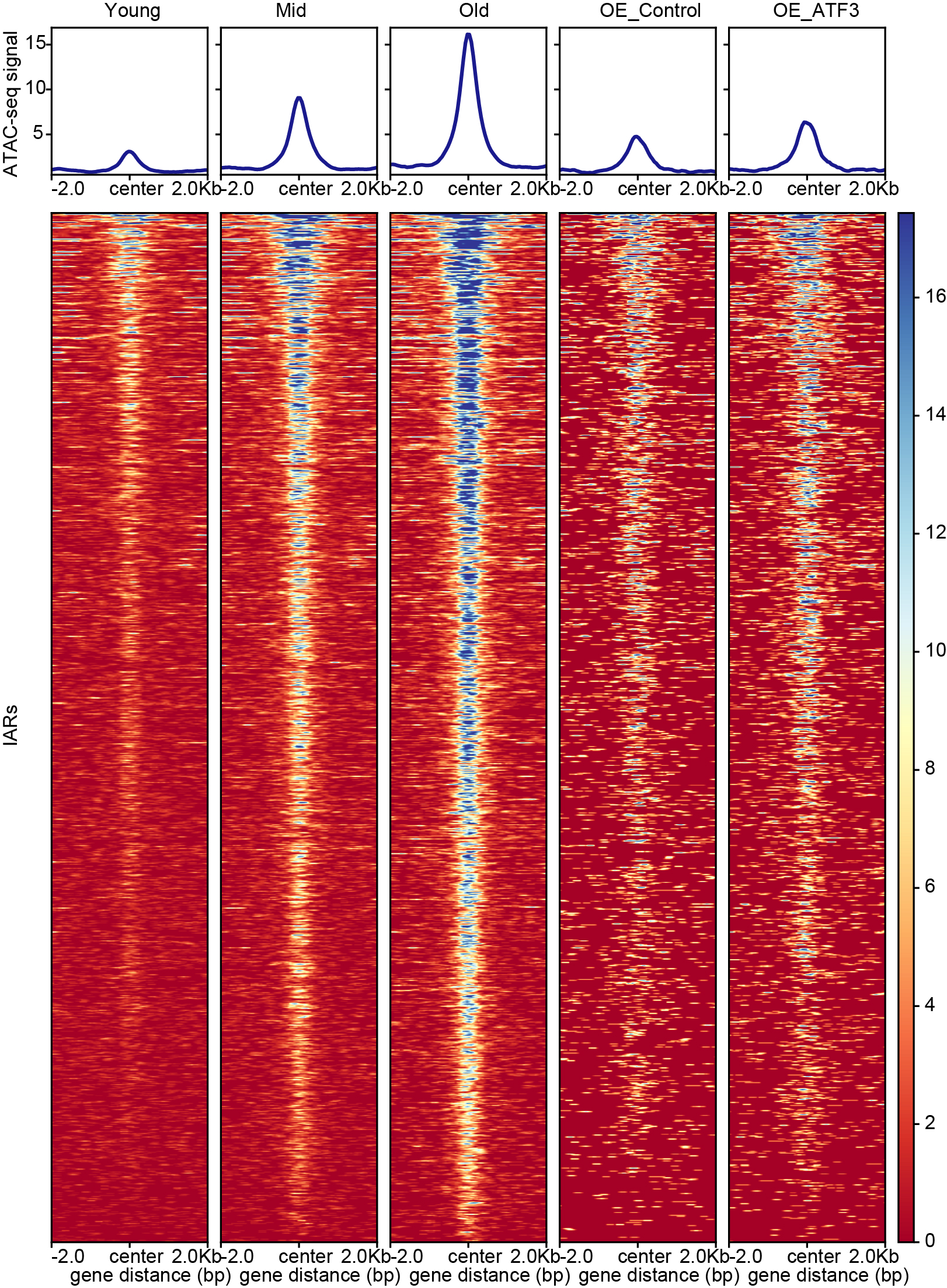
Figure S14.** The ATAC-seq signals at IARs after overexpression of ATF3. ATF3 overexpression was performed in proliferating HUVECs that were harvested at PD14.

**
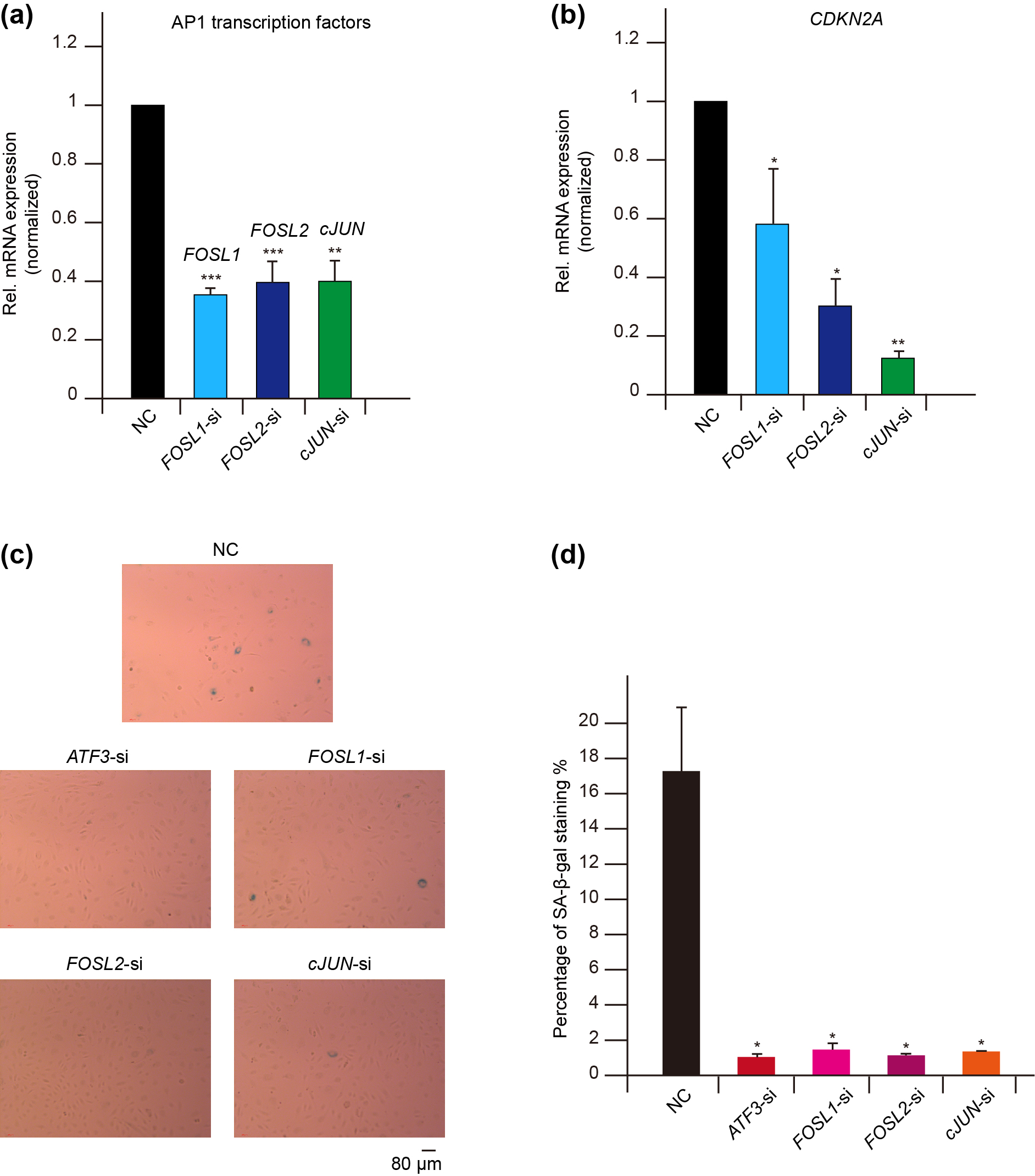
Figure S15.** The impact of AP1-family members to senescence. (a) RT-qPCR analysis for the expression of *FOSL1*, *FOSL2* and *cJUN* after knockdown with corresponding siRNA. NC, negative control siRNA. (b) RT-qPCR analysis for the expression of *CDKN2A* after siRNA knock-down. The cycle threshold (Ct) was normalized with *ACTB*. (c) SA-β-gal staining assay shows the difference of senescence phenotypes in AP1-family members knock-down with siRNA and in control HUVECs. NC, negative control siRNA. (d) Statistical comparison of SA-β-gal positive cells in (c). The error bars represent the s.d. obtained from triplicate independent experiments. Two-tailed, unpaired Student’s t-tests were performed. *P < 0.05, **P < 0.01, ***P < 0.001.

**Supplementary Tables**

Table S1. Primers used in ChIP-qPCR

| Name | Primer sequence |
| --- | --- |
| *LAMB3*-e1 | For: 5’-CAAAGGGAACTGGCCTTATCT-3’ |
|  | Rev: 5’-CTGTGGTCTGGTGAACTCTTAC-3’ |
| *LAMB3*-e2 | For: 5’-CCATAAACAGCCCAAACTCAATAA-3’ |
|  | Rev: 5’-CCAAGTGTGGGAATGAGAAGA-3’ |
| *LAMB3*-e3 | For: 5’-AGTCCGACCTTTATGACCTTTG-3’ |
|  | Rev: 5’-AGCTGTATGTCTCTCTCCTCTC-3’ |
| *LAMB3*-e4 | For: 5’-CTATGGGCACAGATGGAGAAG-3’ |
|  | Rev: 5’-CCTGTTGGTATGAAGGAGGAAA-3’ |
| *CD44*-e1 | For: 5’-CTTCCCACTTCAGTGCTTTCT -3’ |
|  | Rev: 5’-GCACCTTCTCTTCCAGTTCTT-3’ |
| *CD44*-e2 | For: 5’-ACCTGTAGAGAGGGACAGAAA-3’ |
|  | Rev: 5’-TTGTAGGGTTGTGGTGAAGAC-3’ |
| *CD44*-e3 | For: 5’-TCTGGGCCTCAGTTTCTTTATC-3’ |
|  | Rev: 5’-TGCCTCAGTATACAAGTGCTTAG-3’ |
| *CD44*-e4 | For: 5’-CTAGGCTACGATTTGGACTCAG -3’ |
|  | Rev: 5’-CAACTGGAGCATGAAGCTTTG-3’ |
| *CD44*-e5 | For: 5’-TGGCAGAGGAGAGAAGTAGAA-3’ |
|  | Rev: 5’-TGGTCAGATGACAGCTTGATAAT-3’ |
| *CD44*-e6 | For: 5’-TGAAGGAGGAGGAAGTTGTAATG-3’ |
|  | Rev: 5’-CCATCACATGGAAGAGAGTAGAG-3’ |
| *CD44*-e7 | For: 5’-GTCCCAGGTATGCACATCAA-3’ |
|  | Rev: 5’-CCTGCCACCCAAGATGAAATA-3’ |
| *CD44*-e8 | For: 5’-AAACAGTTCTCCATACCCTCAC-3’ |
|  | Rev: 5’-CCCAATGCACAGCCTAGAA-3’ |
| *FGF5*-e1 | For: 5’-GCAGTAGCTGGCTGAGTATTT -3’ |
|  | Rev: 5’-AGTCTGCCTGTTTGGGATTT -3’ |
| *FGF5*-e2 | For: 5’-CCTAGTTGGGCTACATCTGAAA-3’ |
|  | Rev: 5’-TGTGTGCCTATTGCCCTATC-3’ |
| *FGF5*-e3 | For: 5’-GCAGGAAAGTTTGAGAACATGG-3’ |
|  | Rev: 5’-ACCAGACTCACAGCTCAAATC-3’ |

Table S2. Primers used in RT-qPCR

| Genes | Primer sequence |
| --- | --- |
| *CD44* | For: 5’-CTGCCGCTTTGCAGGTGTA-3’ |
|  | Rev: 5’-CATTGTGGGCAAGGTGCTATT-3’ |
| *FGF5* | For: 5’-CACTGATAGGAACCCTAGAGGC-3’ |
|  | Rev: 5’-CAGATGGAAACCGATGCCC-3’ |
| *LAMB3* | For: 5’-CCAAAGGTGCGACTGCAATG-3’ |
|  | Rev: 5’-AGTTCTTGCCTTCGGTGTGG-3’ |
| *CDKN1A* | For: 5’-TGTCCGTCAGAACCCATGC-3’ |
|  | Rev: 5’-AAAGTCGAAGTTCCATCGCTC-3’ |
| *CDKN2A* | For: 5’- CCAACGCACCGAATAGTTACG-3’ |
|  | Rev: 5’- GCGCTGCCCATCATCATG-3’ |
| *ACTB* | For: 5’-CATGTACGTTGCTATCCAGGC-3’ |
|  | Rev: 5’-CTCCTTAATGTCACGCACGAT-3’ |
| *ATF3* | For: 5’-CCTCTGCGCTGGAATCAGTC-3’ |
|  | Rev: 5’-TTCTTTCTCGTCGCCTCTTTTT-3’ |
| *FOSL1* | For: 5’-CAGGCGGAGACTGACAAACTG-3’ |
|  | Rev: 5’-TCCTTCCGGGATTTTGCAGAT-3’ |
| *FOSL2* | For: 5’-CAGAAATTCCGGGTAGATATGCC-3’ |
|  | Rev: 5’-GGTATGGGTTGGACATGGAGG-3’ |
| *cJUN* | For: 5’-TCCAAGTGCCGAAAAAGGAAG-3’ |
|  | Rev: 5’-CGAGTTCTGAGCTTTCAAGGT-3’ |

Table S3. AP1-family members small interfering RNA (siRNA) sequences

| Name | Sequence | |
| --- | --- | --- |
|  | sense（5'-3'） | antisense（5'-3'） |
| si-ATF3-1 | GAGGCGACGAGAAAGAAAUTT | AUUUCUUUCUCGUCGCCUCTT |
| si-ATF3-2 | GCCGAAACAAGAAGAAGGATT | UCCUUCUUCUUGUUUCGGCTT |
| si-FOSL1-1 | CUAGCACAAUUUGCACUAATT | UUAGUGCAAAUUGUGCUAGTT |
| si-FOSL1-2 | GCUCAUCGCAAGAGUAGCATT | UGCUACUCUUGCGAUGAGCTT |
| si-FOSL2-1 | GAACCUCGUCUUCACCUAUTT | AUAGGUGAAGACGAGGUUCTT |
| si-FOSL2-2 | CCUGCAGAAGGAGAUUGCUTT | AGCAAUCUCCUUCUGCAGGTT |
| si-cJUN-1 | GCAAAGAUGGAAACGACCUTT | AGGUCGUUUCCAUCUUUGCTT |
| si-cJUN-2 | UCAUGCUAACGCAGCAGUUTT | AACUGCUGCGUUAGCAUGATT |
| Negative control siRNA | UUCUCCGAACGUGUCACGUTT | ACGUGACACGUUCGGAGAATT |
